# Computational approaches to predict bacteriophage–host relationships

**DOI:** 10.1093/femsre/fuv048

**Published:** 2015-12-09

**Authors:** Robert A. Edwards, Katelyn McNair, Karoline Faust, Jeroen Raes, Bas E. Dutilh

**Affiliations:** 1 Department of Computer Science, San Diego State University, 5500 Campanile Dr., San Diego, CA 92182, USA; 2 Department of Marine Biology, Institute of Biology, Federal University of Rio de Janeiro, CEP 21941-902, Brazil; 3 Division of Mathematics and Computer Science, Argonne National Laboratory, 9700 S. Cass Ave, Argonne, IL 60439, USA; 4 Department of Microbiology and Immunology, Rega Institute KU Leuven, Herestraat 49, 3000 Leuven, Belgium; 5 VIB Center for the Biology of Disease, VIB, Herestraat 49, 3000 Leuven, Belgium; 6 Laboratory of Microbiology, Vrije Universiteit Brussel, Pleinlaan 2, 1050 Brussels, Belgium; 7 Theoretical Biology and Bioinformatics, Utrecht University, Padualaan 8, 3584 CH, Utrecht, the Netherlands; 8 Centre for Molecular and Biomolecular Informatics, Radboud Institute for Molecular Life Sciences, Radboud University Medical Centre, Geert Grooteplein 28, 6525 GA, Nijmegen, the Netherlands

**Keywords:** phages, viruses of microbes, metagenomics, co-occurrence, CRISPR, oligonucleotide usage

## Abstract

Metagenomics has changed the face of virus discovery by enabling the accurate identification of viral genome sequences without requiring isolation of the viruses. As a result, metagenomic virus discovery leaves the first and most fundamental question about any novel virus unanswered: What host does the virus infect? The diversity of the global virosphere and the volumes of data obtained in metagenomic sequencing projects demand computational tools for virus–host prediction. We focus on bacteriophages (phages, viruses that infect bacteria), the most abundant and diverse group of viruses found in environmental metagenomes. By analyzing 820 phages with annotated hosts, we review and assess the predictive power of *in silico* phage–host signals. Sequence homology approaches are the most effective at identifying known phage–host pairs. Compositional and abundance-based methods contain significant signal for phage–host classification, providing opportunities for analyzing the unknowns in viral metagenomes. Together, these computational approaches further our knowledge of the interactions between phages and their hosts. Importantly, we find that all reviewed signals significantly link phages to their hosts, illustrating how current knowledge and insights about the interaction mechanisms and ecology of coevolving phages and bacteria can be exploited to predict phage–host relationships, with potential relevance for medical and industrial applications.

## Abstract (precis)

New viruses infecting bacteria are increasingly being discovered in many environments through sequence-based explorations. To understand their role in microbial ecosystems, computational tools are indispensable to prioritize and guide experimental efforts. This review assesses and discusses a range of bioinformatic approaches to predict bacteriophage–host relationships when all that is known is their genome sequence.

## INTRODUCTION

Until recently, viruses could only be identified by using culture-based methods. For phages, i.e. viruses that infect Bacteria or Archaea, and that constitute the majority of the global virosphere, isolation by plaquing on a bacterial lawn has been the mainstay of viral identification. Plaque assays involve growing the host bacteria with phages on an agar plate and observing plaques, clear areas where the phages killed the host bacteria, and where the phage can be isolated (Lederberg and Lederberg 1953). However, growing phages to high enough titers to observe a plaque may be experimentally challenging, since phages may require appropriate conditions to grow, such as chemical supplements, temperature and specific growth media (Clokie and Kropinski 2008). Moreover, if an infecting phage does not immediately lyse the bacteria it infects, lysogens may arise that are resistant to subsequent infection, leading to anything from cloudy plaques to a complete absence of physical signs of infection (Hanna *et al.*^2012^).

Phages and their hosts are coevolving in a constant arms race. A successful infection of a prokaryotic host cell by a phage will eventually kill that host, so there is a strong selective pressure for the host to evolve resistance to the phage. However, the development of resistance by a host will render a phage unable to infect it, and since the phage is an obligate parasite that cannot proliferate without infecting a cell, there is also a strong selective pressure for the phage to remain counter-adapted (Van Valen 1973; Hyman and Abedon 2010). Many of the host proteins exploited by phages in their life cycle are essential for bacterial growth or beneficial under some circumstances (Meaden, Paszkiewicz and Koskella 2015). Temporal and spatial heterogeneity of environments may thus partially explain how phage and bacteria can coexist without one outcompeting the other (Brüssow 2013; Koskella 2014). Moreover, the arms race is not just a bipartite struggle: the phages are also in a battle with each other to dominate the host, consume its resources and create phage progeny.

Molecular and ecological coevolutionary processes shape phage and bacterial genomes and leave signals in their genome sequences that allow us to predict phage–host interaction. Every step of the phage life cycle is susceptible to mutations that will alter the balance in the phage–host relationship (Labrie, Samson and Moineau 2010). The very first interaction between phage and host involves the binding of the phage to receptor molecules on the surface of the host cell. These receptors are a main candidate for mutations by the bacteria that render the phage inactive. In coculture experiments of phages and bacteria, bacterial mutants that alter the phage receptor rapidly take over the population (Perry, Barrick and Bohannan 2015). Bacteria harboring mutations in many different surface-based receptors have been identified (Chaturongakul and Ounjai 2014). However, the phage receptor molecules are also used by the bacteria for other purposes such as metabolite transport or cell–cell interaction, and mutation of the associated genes may have a negative impact on the fitness of the bacteria in their natural environment. Thus, by mutating receptor molecules the bacteria may be spared from infection in the presence of the phage, but are outcompeted when phages are absent.

Before the phage DNA can be injected into the bacterial cell, there are structural changes that occur in both phage and host. Mutations that prevent these structural changes will also negatively impact phage entry into the host cell, but again at a potential cost to the fitness of the host (Mahony and van Sinderen 2012). As the phage DNA enters the cell, it is most susceptible to interventions that will disrupt the phage replication lifecycle. For instance, clustered regularly interspaced short palindromic repeats (CRISPRs) are a mechanism of acquired bacterial immunity to phages that recognizes and memorizes short subsequences from the genome of the viral invader (Barrangou *et al.*^2007^). The CRISPR system generates short RNA oligonucleotides that, upon reinfection, bind to invading phage DNA and result in the degradation of the phage genome sequence. Host restriction modification systems also attack incoming, unmodified phage DNA, chopping it into fragments that are then degraded (Edwards, Helm and Maloy 1999). Phages that are already present in a host cell can also act to prevent the DNA of competing phages from entering the cell, in a mechanism known as superinfection exclusion (Ebel-Tsipis and Botstein 1971; Susskind, Botstein and Wright 1974).

The step where the genomes of phage and host interact can also be wrought with difficulty for a phage. If the host acquires a mutation in the genomic site where a lysogenic phage typically integrates, either integration or excision could be affected (Smith *et al.*^2010^). Moreover, many phages contain DNA-binding proteins that presumably act as repressors or activators of transcription. These may include potential ‘phage attack modules’ that could enable a phage to remain integrated into a host while that host is attacked by other phages. It has been proposed that some of these proteins act not to control the phage that carries them, but instead to mitigate gene expression of secondarily invading phages (Edwards, Olsen and Maloy 2002). By repressing gene expression, a resident phage could trick a superinfecting competitor into maintaining lysogeny before it has even entered the lysogenic state. This could be brought about by repressing the expression of Integrase that is also usually required for both integration and excision, and thereby preventing integration of the competitor into the bacterial genome. Finally, lysis can also be a target of the arms race, protecting the host cell from bursting and ending the life cycle of the phage in its final stage (Susskind, Wright and Botstein 1974).

In recent years, the introduction of high-throughput DNA sequencing technologies has uncoupled virus discovery from virus isolation. Metagenomics, the untargeted shotgun sequencing of DNA or RNA isolated directly from the environment, is increasingly identifying viral sequences in every imaginable ecosystem (Edwards and Rohwer 2005; Mokili, Rohwer and Dutilh 2012). This approach of sequencing a whole viral community at once allows environmental viruses to be examined without culturing and thus avoids any culturing-associated biases. Metagenomic studies revealed that naturally occurring viral sequences frequently lack detectable homologs in the public databases, highlighting the vast sequence diversity of the virosphere. While these developments greatly accelerate the speed of virus discovery on the way to our grand goal of characterizing the viral sequence space (Dutilh 2014), they come with the disadvantage that a viral genome by itself is little more than a string of nucleotides (Canuti and van der Hoek 2014), especially if it concerns a novel virus with no significantly detectable homologs in the database. Thus, metagenomic virus discovery leaves even the most fundamental question about any novel virus unanswered: What is its host? The direct link with the host, which was available in culturing-based virus discovery, has been lost.

Hand in hand with the advances in sequencing technologies, developments in bioinformatics have facilitated interpretation of the large-scale datasets associated with metagenomics. Traditionally, short metagenomic sequencing reads were analyzed one by one, for example, by aligning them to the annotated sequences in reference databases and allowing them to be individually characterized. However, this ‘read-mapping’ approach depends on the availability of close sequence homologs in the reference database that is problematic for phages, a notoriously understudied and undersequenced component of the global sequence space (Mokili, Rohwer and Dutilh 2012). A promising alternative approach is metagenome assembly that merges short sequencing reads into longer contigs, facilitating downstream analyses including more reliable phage identification and phage–host association. Cross-assembly of different metagenomes extends this approach to incorporate data from many samples (Dutilh *et al.*^2012^). Cross-assembly enables the identification of genomic entities shared between different samples, which also facilitates binning and assembly of genomes (Dutilh *et al.*^2014^). Finally, fosmid cloning of community DNA is an alternative approach that allows the accurate identification of long phage contigs (Mizuno *et al.*2013a,b). Thus far, metagenomic analysis has revealed the genomes of many new phages and unexpected distributions of known ones (Minot *et al.*^2011^, ^2013^; McCallin *et al.*^2013^; Mizuno *et al.*2013b; Dutilh *et al.*^2014^; Aziz *et al.*^2015^).

While invaluable in the analysis of shotgun metagenomic datasets, identifying the genome sequence of a novel phage is only the first step towards understanding its role in the microbial ecosystem. None of the metagenomic approaches outlined above identify the host of a newly discovered phage. Traditional techniques like plaque assays, newer techniques like single celled genomes, and myriad other experimental approaches are available to measure phage–host relationships (Box 1), but these methods frequently require the availability of the phages as cultured isolates. To fully exploit the power of uncultured metagenomics for understanding naturally occurring phages, we review and compare computational approaches for sequence-based prediction of phage–host relationships. These signals include cooccurrence of phages and hosts across environments, genetic homology and exact matches between phage and host genes, the presence of bacterially encoded CRISPR spacers in the phage genomes, and correlations in nucleotide usage profiles (see Table 1). This work has important implications for understanding the natural diversity of phages, the life cycle and coevolution of phages and their hosts, designing experiments to investigate phage–host interactions, inference of phage–bacterial cross-infection networks (Weitz *et al.*^2013^), and investigation of the potential role of phages in horizontal gene exchange, the spread of virulence factors and the proliferation of antibiotic resistance among bacteria (Modi *et al.*^2013^).

**Table 1.** Computational signals to identify bacteriophage–host relationships. The column ‘Performance’ shows for how many of the 820 phages in our benchmarking dataset we could correctly predict the host species (see Fig. 4).

Signal category	Explanation and approach	Performance	Comments
Abundance profiles	Phages can only thrive in an environment if their host is also present. Phage and bacterial abundance patterns in metagenomes can be used to identify their association by (lagged) correlation.	Bacterium with the most similar abundance profile is the correct host species for 9.5% of the phages.	The metagenomics protocol affects the sensitivity of detecting phages and bacteria in a sample. Ecological processes such as Kill-the-Winner can lead to non-linear dynamics that confound straightforward correlations. Stratification of samples by environment may improve the performance.
Genetic homology	Genetic homology between phage and bacterial nucleotide and protein sequences may represent sequences that were acquired by a phage during a past infection event.	Top hit is the correct host species for 38.5% and 29.8% of the phages with blastn and blastx, respectively.	This signal depends on a comprehensive reference database to identify which bacteria are most similar to a given phage. Some gene families are more prone to horizontal gene transfer, leading to some genes being more frequently shared.
CRISPRs	Bacteria place a 25 to 75 bp fragment of an infecting phage sequence into CRISPR arrays on their genome. These arrays can be identified and the spacers aligned to phage genomes to detect recent infections. Multiple spacers between a bacterium and a phage enhance the signal.	Bacterium with the most similar CRISPR spacer is the correct host for 15.1% of the phages. Bacterium with the highest number of CRISPR spacers is the correct host for 21.3% of phages.	Only ∼40% of bacteria and ∼70% of archaea encode a CRISPR system, and the spacers in a CRISPR array are rapidly turned over in the environment. Most CRISPR spacers do not match any known sequence, so although this approach is specific (few false positives), it is not very sensitive (many false negatives).
Exact matches	Exact matches between phage and bacterial genomes can represent integration sites, CRISPR spacers, regions of genetic homology or integrated prophages.	Bacterium with the longest exact match is the correct host species for 40.5% of the phages.	Very short exact matches around the length of integration sites do not contain a significant signal as they can occur randomly.
Oligonucleotide profiles	Over time, phages ameliorate their nucleotide composition towards that of the host. This reflects intracellular nucleotide pools, codon usage and tRNA availability, and restriction-modification systems.	Bacterium with the most similar 4-mer or codon usage profile is correct host species for 17.2% or 10.4% of phages, respectively.	Contrary to this signal, it has often been observed that prophages have a different nucleotide usage profile than the surrounding host genome. Some phages carry tRNA genes to alter the typical host codon usage profile. GC content is a ID measure that does not have a lot of discriminatory power.

Box 1. **Experimental approaches to predict phage–host relationships.**

*Spot assays and plaque assays*

Some experimental approaches to identify which phage infects which bacterium rely on relatively low throughput, time intensive infection assays. The most sensitive tools are spot assays (Middelboe, Chan and Bertelsen 2010). In spot assays, phage isolates are spotted on a bacterial ‘lawn’, consisting of a single bacterial strain grown in a top layer of agar. Typically, the agar is prepared at a lower than normal concentration to allow the phages to spread. If the spotted phage infects and lyses the bacterium, a clearing in the lawn, or ‘plaque’ will arise, indicating lysis of the bacterial cells. Because spot assays combine a cultured host lawn with isolated phages, they are less suitable for investigating environmental phages that typically occur in diverse communities. To overcome this limitation, plaque assays can be used, where a total phage isolate is applied to the host lawn, for example derived from an environmental sample. Dilution series of the phage isolate are often used to create plaques resulting from individual phage clones that may be sampled and analyzed further, including DNA isolation and sequencing.

 Requirements: spot assays require pure culture of both the bacteria and the phage. Plaque assays require pure culture of the bacteria but can use environmental phages.

*Liquid assays*

Liquid assays are a more parallelizable approach to measure the effect of lytic phages on bacterial growth. Bacterial growth in liquid culture is monitored by measuring optical density (OD) or change of a redox dye such as tetrazolium. After addition of a phage isolate, a reduction in the growth curve (relative to a control) indicates that the phage infects and lyses the bacteria. The main drawback of using OD as a readout is that bacterial cell debris resulting from lysis may obscure the measured values, making this method rather unreliable.

 Moreover, measuring a single endpoint value may lead to less sensitive measurements. A recently developed assay exploits the Omnilog platform to circumvent these problems (Henry *et al.*^2012^). The combination of a purple tetrazolium redox dye and the recording of complete bacterial growth curves make this approach more sensitive than liquid assays that use endpoint OD measurements (Henry *et al.*^2012^). Like spot assays, liquid assays require the host to be culturable and phage isolate to be available. Liquid assays can also be used as endpoint assays if the phage genome is known. By using real-time or semiquantitative PCR, amplification of a potential host can be detected in a liquid assay even if the OD does not drop.

 Requirements: liquid assays require pure culture of both the bacteria and the phage.

*Viral tagging*

Viral tagging involves fluorescent labeling of phages followed by adsorption of the phages to host cells if interest, sorting out the host cells that were ‘tagged’ by a fluorescent phage by using a flow cytometer. Next, the viral DNA of adhering phages can be sequenced to identify them (Mosier-Boss *et al.*^2003^; Deng *et al.*^2012^, ^2014^). This technique can be used to measure the presence of phages for a specific host if it is available in pure culture, but it is possible to analyze environmental phages by labeling the total phage fraction in a sample (Mosier-Boss *et al.*^2003^; Deng *et al.*^2012^, ^2014^). Importantly, viral tagging may only measure phage adsorption to the bacterial cell, which does not necessarily result in productive infection and lysis (Deng *et al.*^2012^), for example, in temperate phages but also in possible cases where the phage adheres to a bacterial cell but cannot actually infect it.

 Requirements: viral tagging requires a pure culture of the bacteria but can apply labeled environmental phages.

*Microfluidic PCR*

Individual environmental bacteria can be probed for viruses by using microfluidic digital PCR (Tadmor *et al.*^2011^). In this approach, individual bacterial cells from an environmental sample are sorted out into the tiny reaction chambers of a microfluidic array panel. Some of the chambers may contain a bacterial cell together with an adhering or infecting phage. This is assessed by using PCR, where primers for a bacterial marker gene are combined with primers for a phage marker gene, and then applied to the array. While for bacteria, nearly universal primer sets exist that target the 16S rRNA taxonomic marker gene, universal markers do not exist for phages however signature genes are available for many groups of phage (Dwivedi *et al.*^2012^). Reaction chambers containing both a bacterial and a viral fluorescence signal are then selected and the amplification products are sequenced to identify the bacteria and phage by their sequences.

 Requirements: microfluidic PCR can screen environmental bacteria and phages, but depends on PCR primers targeting a marker gene. These primers can be designed based on metagenomic sequencing data.

*PhageFISH*

Fluorescence *in situ* hybridization (FISH) is commonly applied to microscopically identify microbial cells by hybridizing specific fluorescent probes to their rRNA. Because phages do not contain an abundant RNA hybridization target such as the ribosome, phageFISH modifies this approach by using longer hybridization probes and a catalyzed reported deposition step, allowing intracellular and free viruses to be sensitively visualized (Pernthaler, Pernthaler and Amann 2002; Allers *et al.*^2013^).

 Requirements: phageFISH can screen environmental bacteria and phages, but requires their sequences to design FISH probes. These probes can be designed based on metagenomic sequencing data.

*Single cell sequencing*

Single cell sequencing is an approach where total DNA from a single microbial cell is amplified and sequenced (Lasken and McLean 2014). Given that phage DNA can occur within a host cell, single cell genomics also provides an avenue for identifying phage–host interactions. By screening single cell genome sequences from the marine environment, phages that infect bacterial isolates with no previously identified host were found (Roux *et al.*^2014^; Labonté *et al.*^2015^).

 Requirements: single cell sequencing can screen bacteria and phages directly from the environment, although it should be noted that without prior sequence-dependent screening by PCR or FISH, this approach is biased towards the most abundant environmental bacteria and phages.

*Hi-C sequencing*

Hi-C sequencing is a recently developed technology that measures physically proximal DNA sequences, such as the phage and host genomes present within a single host cell. In this approach, total DNA is first cross-linked, e.g. with formaldehyde, followed by restriction treatment of the DNA and re-ligation of sequence ends that occurred in physical proximity by using ligation enzymes. In principle, this approach could be applicable to natural, complex communities of microbes and phages. While several pilot experiments with mixtures of microbes have been published (Beitel *et al.*^2014^; Burton *et al.*^2014^; Marbouty *et al.*^2014^), Hi-C has to our knowledge not yet been applied to identify links between natural phages and their hosts.

 Requirements: Hi-C sequencing can be applied to screen environmental bacteria and phages.

## PREDICTIVE POWER OF PHAGE–HOST SIGNALS

We review several computational tools and methods for predicting the host of a given phage, when all that is available are their genome sequences (Table 1). Moreover, to compare the performance of each of the methods reviewed, we assess their predictive power by creating bioinformatics scripts to predict phage–host associations in a set of phages with a known host. We used a benchmarking dataset of 820 complete phage genome sequences and 2698 complete bacterial genome sequences that were downloaded from RefSeq on 25 July 2014 (Pruitt *et al.*^2012^). Host information was extracted from the ‘host’ field of the phage RefSeq record, and phages whose host did not have a completely sequenced genome were removed. This provided 820 phages with 153 different bacterial hosts (Supplementary File 1). For all of the analyses, the DNA sequences, open reading frames and their predicted protein translations were extracted from the RefSeq files for all phage and bacterial genomes. As might be expected, most phages infected well-studied organisms including *Escherichia coli* (101 phages), *Pseudomonas aeruginosa* (68 phages) and *Staphylococcus aureus* (67 phages). To perform comparisons at different taxonomic levels, the predicted hosts were compared with the actual host at the ranks of species, genus, family, order, class and phylum by using the NCBI taxonomy tree. In those cases where multiple hosts were predicted, the prediction was scored as correct if the correct host was among the predictions. All the bioinformatics code used in this work is available online at http://edwards.sdsu.edu/PhageHosts/.

### ROC curves

**Figure 1.**
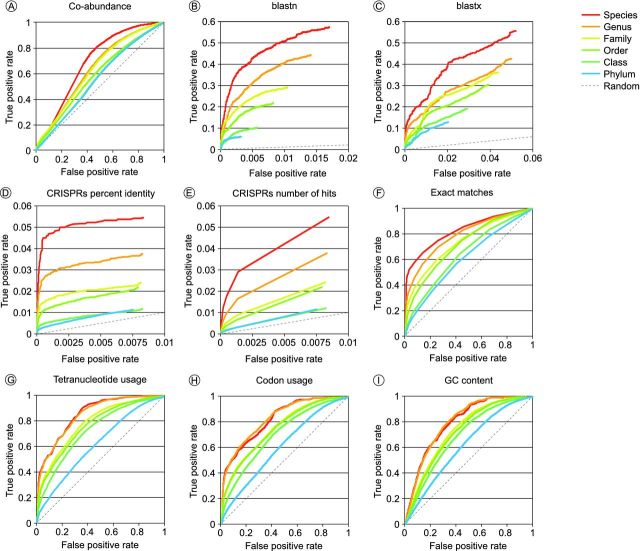
ROC curves displaying the classification accuracy of computational phage–host prediction approaches. (**A**) Pearson correlation of phage and bacterial abundance profiles across environments; (**B**) overall alignment length of blastn hits between phage and bacterial genome sequences; (**C**) number of matching proteins in blastx search of phage DNA to bacterial proteins; (**D**) percent identity of CRISPR spacers aligned to phage genomes; (**E**) number of matching CRISPR spacers in phage genomes; (**F**) length of longest exact match between phage and bacterial genomes; (**G**) Pearson correlation of oligonucleotide usage profiles (tetramers, *k* = 4, for other lengths of *k*, see Fig. S2, Supporting Information); (**H**) similarity in codon usage profiles of phage and bacterial coding regions; (**I**) similarity in GC content between phage and bacterial genomes. Note that in some ROC plots, the TP and FP rates do not continue to FP rate = 1; TP rate = 1. In those cases, we used cutoffs for assignment of a hit.

We use receiver operating characteristic (ROC) curves to display the quality of the predictions made by different approaches. These curves are commonly used in computer science to assess the power of predictive signals. ROC curves are usually plotted in an *x*, *y* plane, where *x* shows the false positive rate (from 0, i.e. no false positives detected, to 1, i.e. 100% of false positives detected), and *y* shows the true positive rate (also from 0 to 1; see Fig. 1 for some examples) (Swets 1996). The idea of an ROC curve is that the cutoff score of a predictive signal is varied from its maximum value to its minimum value. For a given prediction signal, it is expected that phage–host pairs with high scores represent true hits, while phage–host pairs with low scores tend to be incorrect. No phage–host pairs have a score higher than the maximum value, so the ROC plot starts at (*x*, *y*) = (0, 0) i.e. zero true positives and zero false positives detected. As the cutoff score is lowered, more and more phage–host pairs are detected, some being true positives and some being false positives. If the predictor contains a relevant signal, true positives should get detected before false positives, so that from its starting point (0, 0) the ROC curve first goes up along the *y*-axis close to *x* = 0 (no false positives) and reaches a high value on the *y*-axis (many true positives) before going right. As the cutoff score is further lowered to its minimum value, all true and false positives are finally included, and the ROC curve reaches (*x*, *y*) = (1, 1). It should be noted that the ROC curve does not require us to choose a ‘trusted’ cutoff value, because the curve displays the behavior of the entire prediction signal. Because the ROC curve is based on the rate of accumulation of true and false positives, the relative number of either is also not important. The line *x* = *y* is an important line in the ROC curve, as it displays the performance of a random, indistinctive predictor that selects true positives and false positives with an equal probability.

### Abundance profiles

The genomes of phages and their hosts are linked in time and space. This is not only the case for temperate phages that are integrated into the genome of their bacterial host, but also for lytic phages that depend on their host for survival and proliferation. Thus, we only expect to find phages in an environment if their host is also present, providing a link between the genome sequences of phages and their hosts that can be exploited. Metagenomes represent snapshots of natural communities at a certain moment and in a given location, and the abundance patterns of phage and bacterial sequences across metagenomic datasets have been suggested to contain a signal that links the two. Several studies have used this approach to speculate on the host of phages that were identified by their sequence, for example, in metagenomes. For example, Stern *et al.* (2012) showed that for several, but not all of the novel phages they identified in the human gut microbiome, there was a good correlation in abundance with their hosts across metagenomes, suggesting that not all of these phages represented integrated prophages. Reyes *et al*. used the variation of phage and bacterial sequences in metagenomes from the mouse gut to identify potential hosts for the novel phages that they identified. The increase in abundance of two of the five phages coincided with the decrease in abundance of two different bacterial hosts. As the phage abundance waned again, the bacteria recovered, suggesting that they acquired phage resistance mutations (Reyes *et al.*^2013^). They could not, however, speculate on the hosts for the other three phages they identified. Nielsen *et al.* (2014) found that the occurrence of small phage-like gene sets in human gut samples depended on the presence of larger bacterial gene sets, in some cases including known phage–host pairs. In previous work, we clustered the abundance patterns of the bacteriophage crAssphage and 404 potential host bacteria across 151 human fecal metagenomes, showing that this novel bacteriophage clustered deep within a group of *Bacteroidetes* genomes (Dutilh *et al.*^2014^), as did the known *Bacteroides* phages B40-8 and B124-14, providing some evidence that crAssphage may also infect a *Bacteroides* host. The recent Tara Oceans study revealed 1869 positive associations between viral populations and microbial phyla based on correlation analyses. Eight of the identified populations corresponded to phages with a known genome sequence in the Genbank database and for all those cases the correct class of the host was identified, the lowest taxonomic resolution achieved for the host OTUs (Lima-Mendez *et al.*^2015^).

The abundance profiles of phages and hosts are influenced by multiple factors including the burst size of the phage, whether it is virulent or temperate in nature, whether or not the host contains antiviral defense mechanisms, the host range of the phage and the stability or volatility of the phage–host association. Moreover, phage and microbial metagenomes are often isolated and sequenced separately, and sometimes amplified to increase the yield (Rodriguez-Brito *et al.*^2010^). This may affect the perceived abundances and therewith the predicted phage–host relationship. However, depending on the abundance of the virus and the depth of metagenomic sequencing, total community shotgun metagenomes can sometimes be used to simultaneously assess both phage and bacterial sequences in an environment. This is an advantage because it precludes any biases due to differences in sampling or sequencing that might arise when studying microbial and viral metagenomes that were obtained separately. Although phage genomes are very small compared to bacterial genomes, the fraction of phage sequences in total community metagenomes may be quite large. For example, we showed that up to 22% of the metagenomic sequencing reads in total community shotgun metagenomes may be derived from abundant bacteriophages like crAssphage (Dutilh *et al.*^2014^).

To assess the power of environmental coabundance profiles for predicting phage–host interactions, we identified the presence of the bacteria and phages in our benchmarking dataset across 3025 publicly available metagenomes (Meyer *et al.*^2008^). The breakdown of these metagenomes by environmental source is shown in Fig. S1 (Supporting Information). Using custom databases, we applied FOCUS to identify the bacteria present (Silva *et al.*^2014^), and MEGABLAST to identify the phages present in the different metagenomes (Zhang *et al.*^2000^). The abundance profiles across metagenomes of each phage and bacterial genome were compared by using Pearson correlation (Fig. 1A; Table S1, Supporting Information). Because the data sets come from very different environments, most phages and bacteria are mostly absent from most of the metagenomic datasets, leading to sparse abundance profiles with many zero values. Correlating such sparse profiles readily leads to spurious correlations. To account for this, metagenomes were excluded from each pairwise comparison if either the phage or the bacterium was absent from that metagenome, and correlations were only calculated for a phage–host pair if there were >6 metagenomes with non-zero values.

As shown in Fig. 1A, the coabundance measures contain a signal to link phages to their known host, although it is not very strong: this approach correctly identified the species in 12% of the cases (Fig. 4 and Table S1, Supporting Information). Given the success of previous approaches at using coabundance to identify phage–host interactions as described above, this limited performance is perhaps surprising. However, it should be noted that our assays involved a heterogeneous collection of bacterial and phage genome sequences from the RefSeq database, whose abundance was assessed across more than 3000 widely varying metagenomes, while the successful cases all rely on the availability of multiple samples from the same or very similar environments where endemic phages and their hosts interact. Phage communities are highly stratified in space and time (Flores *et al.*^2011^; Flores, Valverde and Weitz 2013; Koskella 2014; Brum *et al.*^2015^; Lima-Mendez *et al.*^2015^). Thus, we expect that the power of coabundance profiles for predicting phage–host relationships will improve as the collection of publicly available metagenomes grows, and further stratification of the metagenomes by environmental parameters becomes possible. Importantly, the availability of multiple samples from similar environments, such as the many samples taken from the human microbiome (Human Microbiome Project Consortium 2012; Nielsen *et al.*^2014^) or the world's oceans (Williamson *et al.*^2008^; Hingamp *et al.*^2013^; Brum *et al.*^2015^; Lima-Mendez *et al.*^2015^), provide the data to sensitively correlate the phages and bacteria found therein. If time-series metagenomic datasets are available, cross-correlation between virus and host abundances might potentially be used to account for time-lagged associations, such as when an outgrowth of a bacterial strain is followed by a peak in the phage that infects that host (Needham *et al.*^2013^; Koskella 2014). The ecology of phage–host interactions, especially predator prey-like dynamics such as Kill-the-Winner (Rodriguez-Valera *et al.*^2009^), can lead to very dynamic changes in abundance of both the phages and their host bacteria that violate the straightforward correlation of their occurrence profiles. In many cases the host may have been lost from a given environment, either through phage infection or (if human/animal samples) through the application of antimicrobial treatments, while the phage may still remain. Finally, the species found in metagenomic datasets cannot always be unambiguously annotated (Hall *et al.*^2015^). The frequent cooccurrence of different bacteria and phages that share large segments of highly conserved sequence may also hamper the detection of relevant coabundance correlations. Thus, not only the increased availability of metagenomic datasets, but also improvements in bioinformatics algorithms for detecting coabundance patterns and annotating metagenomes will improve the power of coabundance profiling for phage–host prediction.

### Genetic homology

Perhaps the most straightforward approach to predict associations between phages and their hosts from their genome sequences is by using sequence similarity searches to identify genetic homology. Homology of phage and bacterial genes indicates recent common ancestry, and a parsimonious explanation for this shared ancestry would be that the phage genome acquired the gene during a recent infection event of that host. Both lytic and temperate phages can mobilize host genetic material and incorporate it into their own genome sequence. Occasionally, these genes provide a benefit, and if they lead to an increase in the phage burst size, natural selection will retain them within the phage genome. One example is auxiliary metabolic genes (Breitbart *et al.*^2007^), such as photosynthesis genes in Cyanophages that are similar to their homologs encoded on the genome of their host (Sullivan, Waterbury and Chisholm 2003; Sharon *et al.*^2011^). Thus, homology between phage and bacterial genes, as identified by sequence similarity searches, has been used to predict phage–bacterial relationships, for example in the gut (Modi *et al.*^2013^; Dutilh *et al.*^2014^).

To assess the power of genetic homology for predicting phage–host associations, we used both nucleotide–nucleotide (blastn) and translated nucleotide–protein (blastx) searches to compare the phage and bacterial genomes, and the proteins they encode (Altschul *et al.*^1990^; Camacho *et al.*^2009^). Nucleotide sequences can change rapidly but still encode the same amino acids because of redundancy in the genetic code. Thus, protein sequences are more conserved in evolution than nucleotide sequences and translated searches are more applicable to distantly related organisms, for example, to bridge the evolutionary gap between the infection event of an ancestral bacterium by an ancestral phage and the present-day sequencing of their descendants. However, we found that nucleotide searches are more accurate than protein searches for predicting the host, as shown in Fig. 1B and C and Tables S2 and S3 (Supporting Information). More than 30% of the hosts were correctly identified at the species level using either similarity search approach, and although approximately the same TP rates were obtained using either search, the nucleotide search had a 3-fold lower FP rate and identified more phage bacterial associations. This suggests that at the greater evolutionary distances covered by translated homology searches, phages are less persistent in their host association, that host switching may occur between closely related phages, or that phages exchange their genetic material via horizontal gene transfer between bacterial genomes. If the phage integrates as a prophage into the bacterial genome, its genes may then undergo rapid amelioration at the DNA sequence level while retaining structure and function at the amino acid level, before being exchanged into another phage genome. These homologs may be recognized at the protein sequence level, even though the DNA sequence has diverged (Lawrence and Ochman 1997; Jensen *et al.*^1998^; Liu *et al.*^2002^; Beumer and Robinson 2005).

### Clustered regularly interspaced short palindromic repeats

Using the CRISPR system, bacteria place a short fragment of an infecting phage genome sequence, typically 25–75 base pairs (bp) long, as a spacer into a CRISPR array, a recognizable repeat region in the bacterial genome (Horvath and Barrangou 2010). This results in a computationally identifiable sequence signature of previous phage–host infections, which has been exploited to identify phage–host interactions in diverse systems including the human microbiome (Stern *et al.*^2012^; Minot *et al.*^2013^), acidophilic biofilms (Andersson and Banfield 2008), cow rumens (Berg Miller *et al.*^2012^), arctic glacial ice and soil (Sanguino *et al.*^2015^), and the marine environment (Anderson, Brazelton and Baross 2011; Cassman *et al.*^2012^). CRISPR spacers commonly have little or no homology to any known sequence, which is thought to reflect the vast uncharacterized sequence space of the virosphere. It was shown in very different natural microbial communities that the spacers in CRISPR arrays are rapidly replaced (Andersson and Banfield 2008; Tyson and Banfield 2008; Pride *et al.*^2011^; Minot *et al.*^2013^). This reflects the ecological dynamics and the constant arms race between bacteria and phages, mediated by the outgrowth of competing strains with different CRISPR arrays in the community and/or the acquisition of new spacers in existing arrays. As a result, the identification of phage–host links by CRISPR spacer matching is likely to be most suitable for detecting recent phage–host interactions, such as within a metagenomic sample where both bacteria and virus components have been sequenced. It should be noted that some bacteria do not encode CRISPRs (Horvath and Barrangou 2010; Reyes *et al.*^2013^), so the approach cannot be applied to those species. About 48 ± 30% and 63 ± 30% of the bacteria and archaea in the various lineages contain a CRISPR-Cas system, respectively (Staals and Brouns 2013). The frequency per lineage differs, ranging from all known species in the *Chlorobi* to complete absence among the *Chlamydiae*. Across all sequenced genomes, the percentages are 39.7% and 69.3% in bacteria and archaea, respectively, although these sequenced genomes form a biased sample of the overall taxonomic diversity (Staals and Brouns 2013).

**Figure 2.**
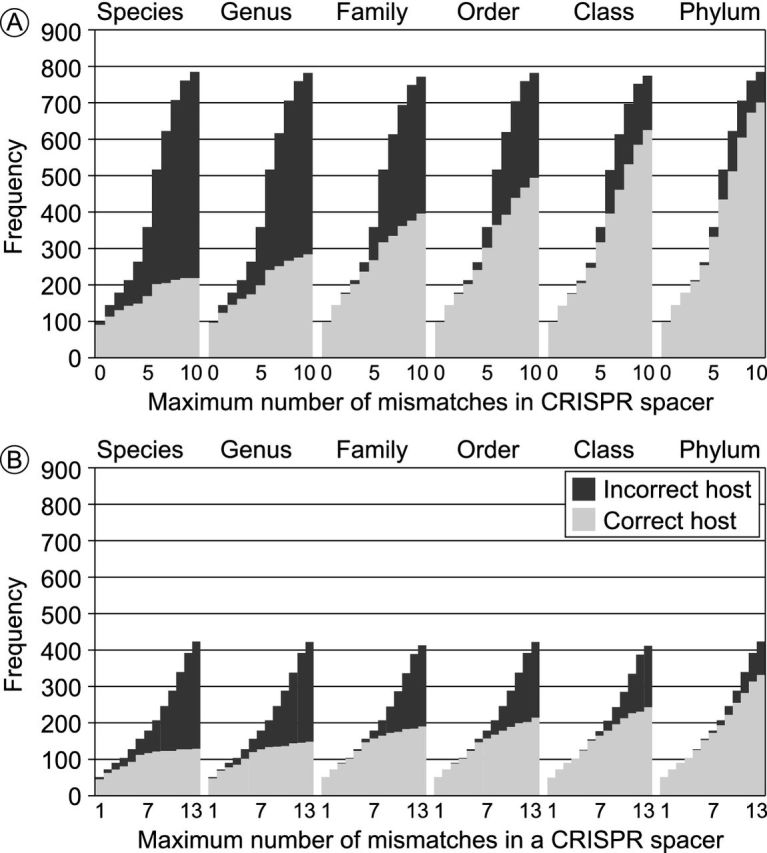
The identification of the number of phages matching a CRISPR spacer in a bacterial genome depends on the number of mismatches between the spacer and the phage genome. (**A**) Number of phages that match at least one CRISPR spacer in a given host; (**B**) number of phages that match at least two CRISPR spacers in a given host. Incorrect host predictions are shown with solid bars and correct host predictions are shown with grey bars.

To assess the power of aligning bacterial CRISPR spacers to phage genome sequences for recognizing phage–host associations, we identified all CRISPR arrays in the 2698 bacterial genomes in our benchmarking dataset, and assessed to what extent the spacers could be aligned to the phage genomes. Several bioinformatics tools have been developed to identify CRISPR spacers in bacterial genomes (Edgar and Myers 2005; Bland *et al.*^2007^; Grissa, Vergnaud and Pourcel 2007a), and spacer sequences have also been collected in publicly accessible databases (Grissa, Vergnaud and Pourcel 2007b; Rousseau *et al.*^2009^). Here, we used Pilercr v1.06 (Edgar and Myers 2005) to extract the 61 552 spacer sequences present in 1066 genomes (i.e. 39.5% of the genomes in our benchmarking dataset), and aligned those spacer sequences to the phage genomes by using blastn (Camacho *et al.*^2009^). Since the default blastn parameters are designed for longer sequences, we adapted the parameters of the search as suggested by the CRISPRTarget tool that identifies the target of CRISPR spacers (Biswas *et al.*^2013^) (i.e. using the blastn-short task, a maximum expect value of 1; a gap opening penalty 10; a gap extension penalty 2; a mismatch penalty 1; a word size 7; and dust filtering turned off). CRISPR spacers were first compared with the viral genomes individually, and for each phage, the bacterium with the best matching CRISPR spacer was predicted to be its host if the spacer had less than a maximum number of mismatches. This approach is very accurate for highly similar CRISPR spacers (Figs 1D and 2A), detecting over 4% of the TPs at very few FPs. The accuracy of this approach for detecting phage hosts strongly depends on the maximum number of mismatches allowed between the CRISPR spacer and the phage genome (Fig. 2A). For example, for phages matching a single CRISPR but allowing two mismatches, 131 of the 178 resulting predictions were correct at the species level (74%). However, there are only few significant hits between CRISPR spacers and phage genomes, so while the phage–host signal contained in CRISPR matches is specific, it is not very sensitive (Figs 1D and 2A; Table S4, Supporting Information). Finding a good CRISPR match is rare, but very relevant if it can be identified. Moreover, this approach will not work for the bacteria and archaea that lack the CRISPR mechanism (Staals and Brouns 2013).

Recent research has shown that the CRISPR-Cas system facilitates priming, a process where non-identical matches between a CRISPR spacer and an invading viral genome lead to the rapid incorporation of new spacers from the same invader (Fineran *et al.*^2014^). This process allows a total of up to 13 mismatches in the sequence of a CRISPR spacer, and it can still be recognized by the CRISPR priming system. Our analysis above showed that for single CRISPR spacers, this level of degeneracy does not contain a significant signal to match phages to their hosts (Fig. 2A). However, because priming leads to the incorporation of multiple CRISPR spacers from the same viral genome into one CRISPR array, we can exploit this additional signal to improve phage–host prediction in some cases. Indeed, phage hosts that are linked by multiple matching spacers give more specific predictions, albeit at a further cost to the sensitivity (Fig. 2B; Table S5, Supporting Information). For example, for phages matching at least two CRISPR spacers, each with at most two mismatches, 64 of the 73 resulting predictions were correct at the species level (88%). The ROC curves show that the number of significant CRISPR spacer hits (Fig. 1E) is a slightly weaker signal than the sequence identity of the optimal hit (Fig. 1D).

### Exact matches

Several molecular mechanisms result in the retention of identical sequences in the genomes of a phage and its host. As discussed above, the conservation of genetic homology and CRISPR spacers both lead to sequence matches between the genomes, in some cases with considerable genetic divergence. Temperate phages that integrate into the genome of their host also provide other sources of exact matching sequences between the genome sequences. First, when the temperate phage is integrated, it can be detected *in silico* with prophage finding tools (Fouts 2006; Lima-Mendez *et al.*^2008^; Akhter, Aziz and Edwards 2012; Roux *et al.*2015a). When matching phages to their host based on sequence information, as reviewed here, the host of a phage that is related to an integrated prophage is readily detected by identifying an (almost) exact match in the bacterial genome corresponding to the full length of the isolated phage. Second, prophage integration sites also contain exact sequence matches between the phage and bacterial genomes, although these are much shorter. The integration of temperate phages into the host genome occurs by homologous recombination, and makes use of recognition sequences on the respective genomes (Campbell 1969), called *attP* (POP') on the phage genome, and *attB* (BOB') on the bacterial genome (Hoess and Landy 1978). These sites consist of flanking DNA (P and P', B and B') that is required for site recognition but which need not share homology, and a common core that is identical between phage and host (O). The size of the common core varies by host and by phage. For example, phage λ uses a tyrosine recombinase and the common core is 15 bp (Hoess and Landy 1978), but in phages that use serine recombinases, the common core ranges from 2 to 12 bp (Smith and Thorpe 2002). It is unlikely that short core sequences, especially those used by serine recombinases, can be distinguished from random sequence matches. However, recombination sites are frequently located adjacent to an integrase gene in the phage genome, and within or near tRNA genes in the bacterial genome (Williams 2002; Julien 2003; Labonté *et al.*^2015^). Thus, the presence of these genes could enhance the confidence of an identified recombination site.

**Figure 3.**
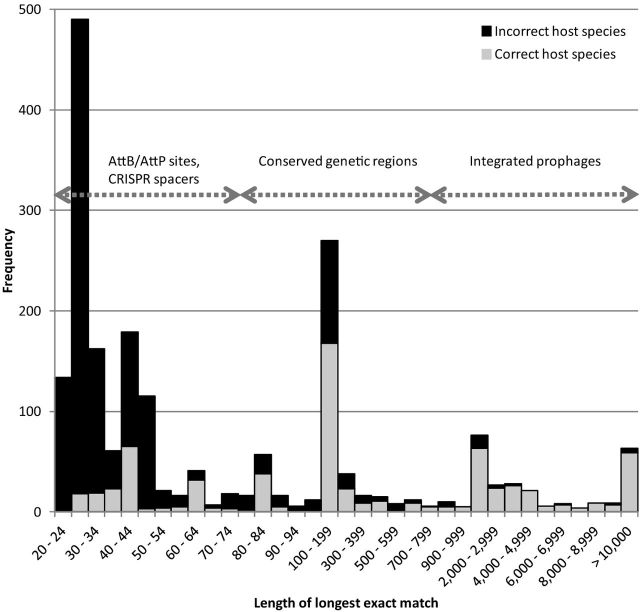
Histogram showing the length of the longest exact match for each phage, divided into correct and incorrect hosts. The approximate size range of several mechanisms leading to exact matches between phage and bacterial genomes are indicated. Note that multiple bacterial genomes can have the same longest exact match with a given phage, in which case they are all included.

To analyze the power of using exact sequence matches between phage and bacterial genomes for *in silico* detection of phage–host interaction, we identified the longest identical sequence between a phage and any bacterial genome sequence. A two-step approach was taken: first, all matching 15-mers were identified between the phage and bacterial genomes and listed sequentially. Next, overlapping identical hits were combined to establish the longest possible match between a phage and bacterial genome sequence, which was used as a signal. The exact matches cover AttB and AttP sites (short), CRISPR spacers (short), conserved genetic regions (short to medium) and integrated prophages (long; see Fig. 3). As might be expected, this approach is highly sensitive, especially for the higher values of the score (bottom left of the ROC plot Fig. 1F), allowing correct prediction of the host species in approximately 40% of the cases (Table S6, Supporting information). Shorter exact matches are less reliable than longer ones because they are more likely to have occurred by random chance. However, there are still several very long exact matches between phages and bacteria that are not annotated as being their host. For example, the two *Burkholderia* phages Bcep176 (44 856 bp) and KS5 (37 236 bp) that are annotated to infect *B. cepacia* and *B. cenocepacia*, respectively, both match chromosome II of *B. multivorans* ATCC 17616 over their full length with just a few mismatches, suggesting that several *Burkholderia* species share this prophage. Similarly, *Staphylococcus* phage SpaA1 (42 784 bp) that is annotated to infect *S. pasteuri* exactly matches the genome of *Bacillus thuringiensis* serovar kurstaki str. HD73 also from the order *Bacillales* (Liu *et al.*^2013^), suggesting that some prophages are conserved between different families from the same orders, as well as between different species from the same genus. These examples are unlikely to reflect sequencing contamination, because the genome sequences of these potential hosts are all complete. Instead, these examples most likely reflect very closely related phages that can infect and integrate into different hosts, or ancestral prophages that integrated into a common ancestor of their current hosts and have not been deleted from the genome.

### Oligonucleotide profiles

Oligonucleotide usage profiles are a way of describing the preferred nucleotide composition of a species at the subsequence level. Oligonucleotide ‘words’, also known as *k*-mers are short subsequences of a specified length that may be extracted from the genome sequence. The profile is a vector that contains the relative frequencies of all *k*-mers in the genome. Phages have been suggested to ameliorate their genomic oligonucleotide usage profile to that of the host they infect (Lawrence and Ochman 1997; Pride *et al.*^2006^). Possible mechanisms are an evolutionary pressure to avoid recognition by host restriction enzymes (Sharp 1986; Pride *et al.*^2006^), or adjustment of codon usage to match the availability of tRNAs during replication in the host cell according to the tRNA adaptation theory (Gouy and Gautier 1982). Thus, oligonucleotide usage profiles might be consistent between phages and their host, providing a signal for computational prediction of phage–host relationships (Roux *et al.*2015b).

In a recent study, tetranucleotide profiles (*k* = 4) were used to extract the sequences of phages with a proposed *Bacteroidales* host from metagenomes (Ogilvie *et al.*^2013^). This study recovered a total of 408 metagenomic fragments with tetranucleotide profiles similar to known *Bacteroidales* phages, many of them with distinct phage-like properties. Two considerations are important when exploiting *k*-mer profiles. First, the length of *k* should be small enough to create a profile that is not too sparse (i.e. it should not contain many zeroes). Longer lengths of *k* may result in highly specific oligonucleotides. For example, 12-mer oligonucleotides have previously been used to define a library of ‘phage words’ that uniquely identify prophages in bacterial genomes (Akhter, Aziz and Edwards 2012). For longer lengths of *k*, the zeroes in the increasingly sparse frequency vectors lead to the possibility of spurious associations when correlating them. The second consideration is the length of the phage and bacterial sequences that are compared that should be as long as possible to obtain a profile that is representative of the genome. Ideally, the entire phage or bacterial genome is used, although this may be challenging when using contigs that were assembled from a shotgun metagenome.

To assess the power of oligonucleotide profiles for predicting phage–host interaction, we calculated *k*-mer profiles of length *k* = 3–8 bp using Jellyfish (Marçais and Kingsford 2011). Because forward- and reverse-complement *k*-mers are counted only once, this vector contains 4*^k^* / 2 values for odd-length *k*-mers, or 2*^k^* + (4*^k^* - 2*k*) / 2 values for even-length *k*-mers, 2*^k^* of which are their own reverse complement. Thus, our frequency vectors contained between 4^3^ / 2 = 32 and 2^8^ + (4^8^ - 2^8^) / 2 = 32896 values. The smallest Euclidean distance between a phage's tetranucleotide usage profile and the profiles of all bacteria was used to identify the potential hosts (other distance measures and oligonucleotide lengths were also tested, see Figs S2 and S3, respectively, Supporting Information). Moreover, we also included two special cases of oligonucleotide usage profiles, the GC content (*k* = 1 bp, a vector containing two values) and codon usage (*k* = 3 bp in frame within the genetic coding regions, a vector of length 64 values). In both these cases, the Euclidean distance of phage's profile to the host's profiles was used to identify the appropriate host.

Based on the ROC plots (Fig. 1G–I), oligonucleotide usage profiles contain a strong phage–host signal, although the correct host could not always be identified as the highest scoring host for a phage. Of all the correlation statistics and lengths of *k*, the Euclidean distance of tetranucleotide profiles provided the strongest signal (Figs S2 and S3). The *k*-mer profiles of length *k* = 3–8 bp predicted between 8% and 17% of the hosts correctly at the species level (Table S7–12, Supporting Information), where longer oligonucleotides are stronger. The percent GC and codon usage predicted approximately 10% of the hosts correctly at the species level (Tables S13 and 14, Supporting Information). GC content has a limited range (from 20% to 80% GC), and apparently this 1D feature is insufficient to discriminate among more than 2000 host genomes.

## DISCUSSION, OUTLOOK AND CONCLUSIONS

**Figure 4.**
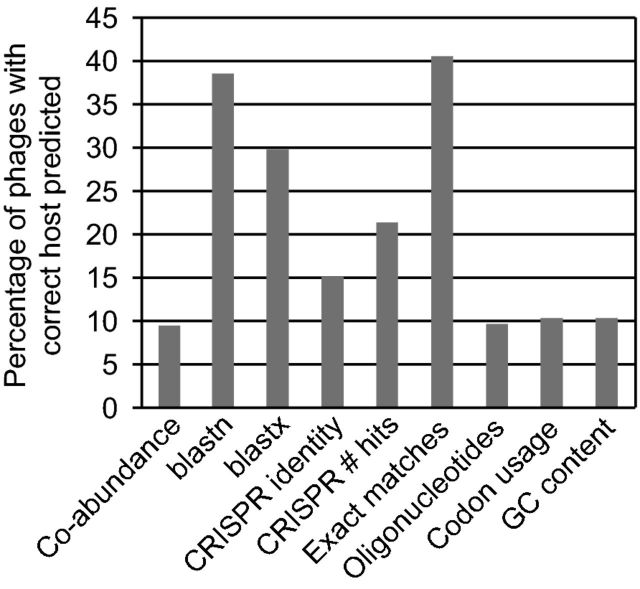
Percentage of phages with a correctly predicted bacterial species among the top scoring hosts using the different computational phage–host prediction approaches. Only the highest scoring bacteria were included, but if multiple top scoring hosts were present, the prediction was scored as correct if the correct host was among the predicted hosts. For details, including the percentage of phages with a correctly predicted host at different taxonomic levels, see Tables S1–18 (Supporting Information).

We have provided an extensive review of computational approaches for predicting phage–host interactions, including occurrence profiles, genetic homology, analysis of CRISPR spacers, exact matches and similarities in oligonucleotide profiles. We used a defined benchmarking dataset of 820 phages with a known host to compare each the methods directly, and Fig. 4 shows, for each method, the percentage of phages whose host species was correctly predicted. The strongest signals included homology-based approaches, including blastn and exact matches in particular. While this result might be expected, it is encouraging to see that the homology-independent approaches also contain a significant predictive signal, providing promise for the prediction of bacterial hosts for completely novel bacteriophages detected in environmental shotgun metagenomes.

Most of the methods predict between 1 and 4 bacteria as the most likely host for a phage (see Tables S1–14, Supporting Information), and in 10%–40% of the cases, this includes the correct host species (Fig. 4). This is much better than random for all prediction signals. If we assign each phage to between one and four randomly predicted hosts, the correct host species is only selected between 1% and 3% of the cases (Tables S15–18; Fig. S3, Supporting Information). Furthermore, it is important to bear in mind that all the computational approaches to predict bacteriophage–host relationships require that at least a significant fraction of the host genome sequence is available as a reference. The exception is abundance profiling, where reliable abundance profiles based on marker genes can also be used. However, the main limitation of that approach is that a sufficiently large number of relatively homogeneous samples are needed. Although microbial genome space is increasingly sampled by sequencing cultured strains, single cells and assembling metagenomes, we still lack genome sequences for most host strains of naturally occurring phages (Wu *et al.*^2009^; Rinke *et al.*^2013^; Dutilh *et al.*^2014^; Brown *et al.*^2015^; Garza and Dutilh 2015).

Studies that focus on computational prediction of phage–host relationships implicitly assume that a virus infects a single host. Indeed, phages can be very host specific, some being used for bacterial strain typing, for example of clinically relevant bacteria. However, there is currently insufficient evidence to make broad generalizations about phage host range in natural populations (Koskella and Meaden 2013). As we have seen above, the short exact matches utilized by Integrase to insert the phage into the host genome are not discriminative of the host, suggesting that a single phage could potentially insert into multiple host genomes. Similarly, other molecular mechanisms might also be used to infect alternative hosts, and some phage genomes might even encode different genes relevant to infecting different hosts. It is perhaps surprising that phages do not appear to infect multiple different hosts to extend their chances of a successful infection. In general, the evolution of virus–host specificity and the related infection mechanisms remain poorly understood, even in clinically important viruses like influenza A that, upon evolving to infect a different host, can cause global epidemics in humans (Taubenberger and Kash 2010). In phages, host specificity is increasingly considered to be highly variable, with some phages being very host specific, infecting a single host or a very narrow host range at best, and others being able to infect multiple host strains. Both theoretical considerations and observations based on large-scale analyses support the idea that some phages may be able to infect multiple hosts (Flores, Valverde and Weitz 2013; Jover, Cortez and Weitz 2013; Koskella and Meaden 2013; Weitz *et al.*^2013^; Chow *et al.*^2014^). For example, a large phage–host network predicted from marine viromes mostly displayed narrow host ranges, but also contained a few phage hubs (Lima-Mendez *et al.*^2015^). A meta-analysis of phage–bacterial interaction networks showed that these networks are globally modular and locally nested, which means that phages from different geographical locations are mostly incompatible, while locally, phages might infect several different hosts (Flores *et al.*^2011^; Beckett and Williams 2013; Flores, Valverde and Weitz 2013). This modularity in the marine phage–host network was found across different oceanic regions (Flores *et al.*^2011^; Flores, Valverde and Weitz 2013; Brum *et al.*^2015^). However, at the level of individual phage isolates, a wide host range has not frequently been observed, probably due to the very rapid specialization of phages to the dominant hosts in their present environment (Koskella 2014). Phages have occasionally been observed to switch or adapt to different hosts (Bertani and Weigle 1953; van de Putte, Cramer and Giphart-Gassler 1980; Liu *et al.*^2002^). Many, but not all, coliphages can also infect *E. coli*'s close relative *Shigella*, and many *Streptomyces* phages exhibit similar broad host ranges among its close relatives*.* In addition, some phages recognize plasmid-borne receptors on the cell surface and can infect any host carrying the plasmid. For example, PRD1 can infect a range of hosts carrying plasmids with P, N or W incompatibility groups (Olsen, Siak and Gray 1974). In our benchmarking dataset based on annotated phage–host associations, each phage is annotated with a single bacterial host, and it should be noted that not all possible bacterial hosts were experimentally tested during the characterization of these phages. While reviewing the various phage–host signals above, we observed that some phage genomes contained strong signals linking them to diverse bacterial hosts, such as genes with high-sequence similarity to very diverse bacteria, possibly reflecting infection of these diverse hosts in recent evolutionary history. It is clear that extended datasets of experimentally measured phage–bacterial infections are needed to definitively answer the question how specific phage–host interactions are, and how rapidly host tropism switches or evolves. Bioinformatics approaches will make a valuable contribution by predicting the most likely candidates for experimental testing.

As new technologies are opening up the potential of identifying viruses without first culturing them, upending the traditional approach for virus discovery, new bioinformatics tools and techniques will be required to direct the experimental work to characterize those viruses. We reviewed currently available approaches for predicting phage–host relationships based on their genome sequences (Table 1). While some signals are stronger predictors than others, we find that all the reviewed signals contain a significant signal linking phages to their hosts (Fig. 1). This shows how advances in biological knowledge and an improved understanding of the interactions between phages and bacteria can be exploited in predictive tools. We expect that this understanding will only improve with the recent increased interest of biologists in bacteriophage research, and that this will lead to promising new ideas for phage–host signals. In turn, these tools can be exploited by phage biologists exploring the virosphere to understand the natural diversity, life cycle, interactions, and coevolution of phages and their hosts. Moreover, by providing rapid *in silico* prioritization of candidates for experimental testing and contributing to experimental design, these new bioinformatics approaches will alleviate and direct experimental efforts by proposing testable hypotheses. Finally, applying these tools on a large scale, they will allow the inference of phage–bacterial cross-infection networks (Weitz *et al.*^2013^; Chow *et al.*^2014^) and support investigations into the potential role of phages in horizontal gene exchange, the spread of virulence factors and the proliferation of antibiotic resistance among bacteria (Modi *et al.*^2013^). Thus, these tools will improve our understanding of virus–host interactions in natural systems, and of the microbial ecology of the environments that are sampled by metagenomics.

## Supplementary Material

Supplementary Data
