# Supplementary material for: Computational approaches to predict bacteriophage–host relationships
Source: FEMS Microbiol Rev. 2015 Dec 9;40(2):258–72. doi: 10.1093/femsre/fuv048 (PMC5831537; doi:10.1093/femsre/fuv048)
Supplement: Supplementary Data [file fuv048_supplementary_data.zip › Supplemental Tables 20150927.docx]

**Computational approaches to predict bacteriophage-host relationships**

Supplemental Tables

*Co-abundance*

Table S1. Host predictions using co-abundance of phages and bacteria across metagenomes as a phage-host signal. Normalized presence of the bacteria and phages in each of 3,205 metagenomes was used to calculate Pearson's *r* between phage and host. For each phage, the bacteria (average: 1.74) with the highest co-abundance correlation were considered as potential hosts.

| Taxonomic level | Correct assignments | Incorrect assignments | Total Assignments | Percent Correct |
| --- | --- | --- | --- | --- |
| species | 100 | 720 | 820 | 12.2 |
| genus | 131 | 689 | 820 | 15.98 |
| family | 182 | 638 | 820 | 22.2 |
| order | 223 | 597 | 820 | 27.2 |
| class | 320 | 500 | 820 | 39.02 |
| phylum | 437 | 383 | 820 | 53.29 |

*Genetic homology*

Table S2. Host predictions using genetic homology between phage and bacterial genomes as a phage-host signal. This comparison use a nucleotide (blastn) search of 820 phage genomes against a database of 2,698 complete bacterial genomes. The blastn parameters were adapted as follows: word size was reduced to 5; maximum E-value 10; the number of reported descriptions was 100; the reward for a match was 1; the penalty for a mismatch was -2; the gap opening penalty was 0; the gap extension penalty was 0. For each phage, the bacteria (average: 1.40) with the longest overall alignment length were considered as potential hosts.

| Taxonomic level | Correct Assignments | Incorrect Assignments | Total Assignments | Percent Correct |
| --- | --- | --- | --- | --- |
| species | 369 | 451 | 820 | 45 |
| genus | 511 | 309 | 820 | 62.32 |
| family | 606 | 214 | 820 | 73.9 |
| order | 668 | 152 | 820 | 81.46 |
| class | 726 | 94 | 820 | 88.54 |
| phylum | 786 | 34 | 820 | 95.85 |

Table S3. Host predictions using genetic homology between phage genomes and bacterial proteins as a phage-host signal. This comparison includes a translated nucleotide – protein (blastx) search of all phage genomes against a database of all proteins from 2,698 complete bacterial genomes. For each phage, the bacteria (average: 1.61) with the highest number of matching proteins were considered as potential hosts.

| Taxonomic level | Correct Assignments | Incorrect Assignments | Total Assignments | Percent Correct |
| --- | --- | --- | --- | --- |
| species | 268 | 552 | 820 | 32.68 |
| genus | 384 | 436 | 820 | 46.83 |
| family | 460 | 360 | 820 | 56.1 |
| order | 549 | 271 | 820 | 66.95 |
| class | 659 | 161 | 820 | 80.37 |
| phylum | 759 | 61 | 820 | 92.56 |

*Clustered regularly interspaced short palindromic repeats (CRISPRs)*

Table S4. Host predictions using percent identity between bacterial CRISPR spacers and phage genomes as a phage-host signal. CRISPR spacers were identified in all bacterial genomes using Pilercr v1.06 and aligned to the phage genomes using blastn with CRISPRTarget parameters (Biswas *et al.*, 2013), i.e. blastn-short, E-value<1; gap opening penalty 10; gap extension penalty 2; mismatch penalty 1; word size 7; and dust filtering turned off. For each phage, the bacteria (average: 3.93) with the highest percent identity with a CRISPR spacer were considered as potential hosts.

| Taxonomic level | Correct Assignments | Incorrect Assignments | Total Assignments | Percent Correct |
| --- | --- | --- | --- | --- |
| species | 127 | 693 | 820 | 15.49 |
| genus | 174 | 646 | 820 | 21.22 |
| family | 255 | 565 | 820 | 31.1 |
| order | 299 | 521 | 820 | 36.46 |
| class | 389 | 431 | 820 | 47.44 |
| phylum | 551 | 269 | 820 | 67.2 |

Table S5. Host predictions using the number of matching CRISPR spacers between phage and bacterial genomes as a phage-host signal. CRISPR spacers were identified and searched as described in Table S4. For each phage, the bacteria (average: 1.34) with the highest number of matching CRISPR spacers were considered as potential hosts.

| Taxonomic level | Correct Assignments | Incorrect Assignments | Total Assignments | Percent Correct |
| --- | --- | --- | --- | --- |
| species | 178 | 642 | 820 | 21.71 |
| genus | 221 | 599 | 820 | 26.95 |
| family | 307 | 513 | 820 | 37.44 |
| order | 360 | 460 | 820 | 43.9 |
| class | 425 | 395 | 820 | 51.83 |
| phylum | 518 | 302 | 820 | 63.17 |

*Exact matches*

Table S6. Host predictions based on exact matches ≥15bp between phage and bacterial genomes. For each phage, the bacteria (average: 2.42) with the longest exact match were considered as potential hosts.

| Taxonomic level | Correct Assignments | Incorrect Assignments | Total Assignments | Percent Correct |
| --- | --- | --- | --- | --- |
| species | 332 | 488 | 820 | 40.49 |
| genus | 413 | 407 | 820 | 50.37 |
| family | 470 | 350 | 820 | 57.32 |
| order | 502 | 318 | 820 | 61.22 |
| class | 569 | 251 | 820 | 69.39 |
| phylum | 653 | 167 | 820 | 79.63 |

*Oligonucleotide usage profiles*

Table S7. Host predictions using Euclidean distance of the profile of all trimer frequencies (*k=*3) in the phage and bacterial genomes. For each phage, the bacteria (average: 1.49) with the most similar oligonucleotide usage profile were considered as potential hosts.

| Taxonomic level | Correct assignments | Incorrect assignments | Total Assignments | Percent Correct |
| --- | --- | --- | --- | --- |
| species | 67 | 753 | 820 | 8.17 |
| genus | 177 | 643 | 820 | 21.59 |
| family | 239 | 581 | 820 | 29.15 |
| order | 301 | 519 | 820 | 36.71 |
| class | 434 | 386 | 820 | 52.93 |
| phylum | 492 | 328 | 820 | 60 |

Table S8. Host predictions using Euclidean distance of the profile of all tetramer frequencies (*k=*4) in the phage and bacterial genomes. For each phage, the bacteria (average: 1.40) with the most similar oligonucleotide usage profile were considered as potential hosts.

| Taxonomic level | Correct assignments | Incorrect assignments | Total Assignments | Percent Correct |
| --- | --- | --- | --- | --- |
| species | 80 | 740 | 820 | 9.76 |
| genus | 204 | 616 | 820 | 24.88 |
| family | 269 | 551 | 820 | 32.8 |
| order | 305 | 515 | 820 | 37.2 |
| class | 457 | 363 | 820 | 55.73 |
| phylum | 524 | 296 | 820 | 63.9 |

Table S9. Host predictions using Euclidean distance of the profile of all pentamer frequencies (*k=*5) in the phage and bacterial genomes. For each phage, the bacteria (average: 1.39) with the most similar oligonucleotide usage profile were considered as potential hosts.

| Taxonomic level | Correct assignments | Incorrect assignments | Total Assignments | Percent Correct |
| --- | --- | --- | --- | --- |
| species | 101 | 719 | 820 | 12.32 |
| genus | 236 | 584 | 820 | 28.78 |
| family | 299 | 521 | 820 | 36.46 |
| order | 347 | 473 | 820 | 42.32 |
| class | 464 | 356 | 820 | 56.59 |
| phylum | 518 | 302 | 820 | 63.17 |

Table S10. Host predictions using Euclidean distance of the profile of all hexamer frequencies (*k=*6) in the phage and bacterial genomes. For each phage, the bacteria (average: 1.29) with the most similar oligonucleotide usage profile were considered as potential hosts.

| Taxonomic level | Correct assignments | Incorrect assignments | Total Assignments | Percent Correct |
| --- | --- | --- | --- | --- |
| species | 106 | 714 | 820 | 12.93 |
| genus | 257 | 563 | 820 | 31.34 |
| family | 329 | 491 | 820 | 40.12 |
| order | 374 | 446 | 820 | 45.61 |
| class | 471 | 349 | 820 | 57.44 |
| phylum | 529 | 291 | 820 | 64.51 |

Table S11. Host predictions using Euclidean distance of the profile of all heptamer frequencies (*k=*7) in the phage and bacterial genomes. For each phage, the bacteria (average: 1.28) with the most similar oligonucleotide usage profile were considered as potential hosts.

| Taxonomic level | Correct assignments | Incorrect assignments | Total Assignments | Percent Correct |
| --- | --- | --- | --- | --- |
| species | 119 | 701 | 820 | 14.51 |
| genus | 270 | 550 | 820 | 32.93 |
| family | 337 | 483 | 820 | 41.1 |
| order | 378 | 442 | 820 | 46.1 |
| class | 480 | 340 | 820 | 58.54 |
| phylum | 538 | 282 | 820 | 65.61 |

Table S12. Host predictions using Euclidean distance of the profile of all octamer frequencies (*k=*8) in the phage and bacterial genomes. For each phage, the bacteria (average: 1.33) with the most similar oligonucleotide usage profile were considered as potential hosts.

| Taxonomic level | Correct assignments | Incorrect assignments | Total Assignments | Percent Correct |
| --- | --- | --- | --- | --- |
| species | 140 | 680 | 820 | 17.07 |
| genus | 284 | 536 | 820 | 34.63 |
| family | 349 | 471 | 820 | 42.56 |
| order | 383 | 437 | 820 | 46.71 |
| class | 499 | 321 | 820 | 60.85 |
| phylum | 556 | 264 | 820 | 67.8 |

Table S13. Host predictions based on similarity of phage and bacterial codon usage profiles. Similarities were calculated as 1 minus the Euclidean distance between the phage and bacterial codon usage profiles. For each phage, the bacteria (average: 17.00) with the most similar codon usage were considered as potential hosts.

| Taxonomic level | Correct Assignments | Incorrect Assignments | Total Assignments | Percent Correct |
| --- | --- | --- | --- | --- |
| species | 85 | 735 | 820 | 10.37 |
| genus | 125 | 695 | 820 | 15.24 |
| family | 162 | 658 | 820 | 19.76 |
| order | 345 | 475 | 820 | 42.07 |
| class | 743 | 77 | 820 | 90.61 |
| phylum | 803 | 17 | 820 | 97.93 |

Table S14. Host predictions based on similarity of the GC content in the coding regions of phage and bacterial genomes. Similarities were calculated as 1 minus the Euclidean distance between the phage and bacterial GC content within coding regions. For each phage, the bacteria (average: 17.00) with the most similar GC content were considered as potential hosts.

| Taxonomic level | Correct Assignments | Incorrect Assignments | Total Assignments | Percent Correct |
| --- | --- | --- | --- | --- |
| species | 85 | 735 | 820 | 10.37 |
| genus | 125 | 695 | 820 | 15.24 |
| family | 162 | 658 | 820 | 19.76 |
| order | 345 | 475 | 820 | 42.07 |
| class | 743 | 77 | 820 | 90.61 |
| phylum | 803 | 17 | 820 | 97.93 |

*Random host assignments*

Table S15. Host predictions using random assignment of 1 host per bacteriophage. Average ± standard error of 100 random samples is listed.

| Taxonomic level | Correct Assignments | Incorrect Assignments | Total Assignments | Percent Correct |
| --- | --- | --- | --- | --- |
| species | 6.45 ± 2.78 | 813.55 ± 2.78 | 820 | 0.78 ± 0.34 |
| genus | 14.5 ± 3.98 | 798.73 ± 4.47 | 820 | 1.78 ± 0.49 |
| family | 33.79 ± 6.4 | 748.01 ± 8.18 | 820 | 4.32 ± 0.82 |
| order | 53.4 ± 7.84 | 753.43 ± 8.3 | 820 | 6.61 ± 0.97 |
| class | 141.69 ± 11.15 | 646.4 ± 12.21 | 820 | 17.97 ± 1.42 |
| phylum | 282.37 ± 13.47 | 535.94 ± 13.45 | 820 | 34.5 ± 1.64 |

Table S16. Host predictions using random assignment of 2 host per bacteriophage. Average ± standard error of 100 random samples is listed.

| Taxonomic level | Correct Assignments | Incorrect Assignments | Total Assignments | Percent Correct |
| --- | --- | --- | --- | --- |
| species | 12.04 ± 3.44 | 807.96 ± 3.44 | 820 | 1.46 ± 0.42 |
| genus | 28.23 ± 5.37 | 784.91 ± 5.98 | 820 | 3.47 ± 0.66 |
| family | 64.25 ± 7.33 | 723.54 ± 9.21 | 820 | 8.15 ± 0.93 |
| order | 103.03 ± 9.53 | 703.5 ± 10.28 | 820 | 12.77 ± 1.19 |
| class | 255 ± 14.58 | 542.18 ± 15.24 | 820 | 31.98 ± 1.84 |
| phylum | 451 ± 13.56 | 367.76 ± 13.68 | 820 | 55.08 ± 1.66 |

Table S17. Host predictions using random assignment of 3 host per bacteriophage. Average ± standard error of 100 random samples is listed.

| Taxonomic level | Correct Assignments | Incorrect Assignments | Total Assignments | Percent Correct |
| --- | --- | --- | --- | --- |
| species | 18.54 ± 4.26 | 801.46 ± 4.26 | 820 | 2.26 ± 0.52 |
| genus | 40.72 ± 6.5 | 772.21 ± 6.7 | 820 | 5 ± 0.8 |
| family | 91.93 ± 8.99 | 700.62 ± 10.06 | 820 | 11.59 ± 1.13 |
| order | 147.08 ± 10.85 | 659.7 ± 10.55 | 820 | 18.22 ± 1.33 |
| class | 346.42 ± 12.51 | 457.58 ± 11.96 | 820 | 43.08 ± 1.51 |
| phylum | 552.96 ± 10.74 | 266.09 ± 10.77 | 820 | 67.51 ± 1.31 |

Table S18. Host predictions using random assignment of 4 host per bacteriophage. Average ± standard error of 100 random samples is listed.

| Taxonomic level | Correct Assignments | Incorrect Assignments | Total Assignments | Percent Correct |
| --- | --- | --- | --- | --- |
| species | 25.22 ± 5.1 | 794.78 ± 5.1 | 820 | 3.07 ± 0.62 |
| genus | 54.33 ± 7.48 | 758.45 ± 7.63 | 820 | 6.68 ± 0.92 |
| family | 119.93 ± 9.72 | 675.39 ± 10.04 | 820 | 15.07 ± 1.21 |
| order | 188.87 ± 11.89 | 618.99 ± 12.16 | 820 | 23.37 ± 1.47 |
| class | 419.2 ± 13.9 | 387.51 ± 13.98 | 820 | 51.96 ± 1.72 |
| phylum | 616.31 ± 10.95 | 202.91 ± 10.9 | 820 | 75.23 ± 1.33 |
