# Supplementary figures and images for: Computational approaches to predict bacteriophage–host relationships
Source: FEMS Microbiol Rev. 2015 Dec 9;40(2):258–72. doi: 10.1093/femsre/fuv048 (PMC5831537; doi:10.1093/femsre/fuv048)

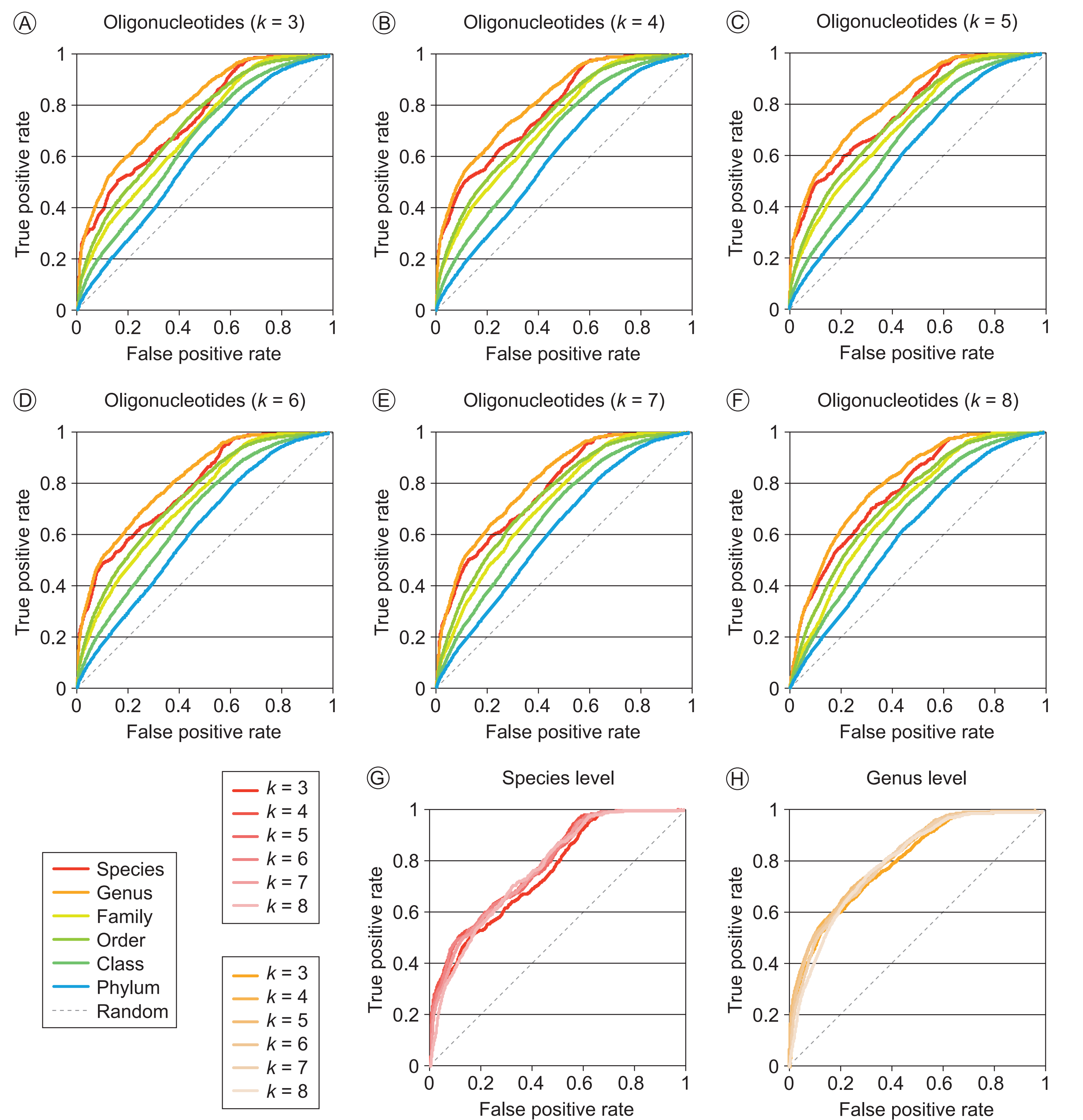

Supplement: Supplementary Data [file fuv048_supplementary_data.zip › SupplFigure_S1.pdf]

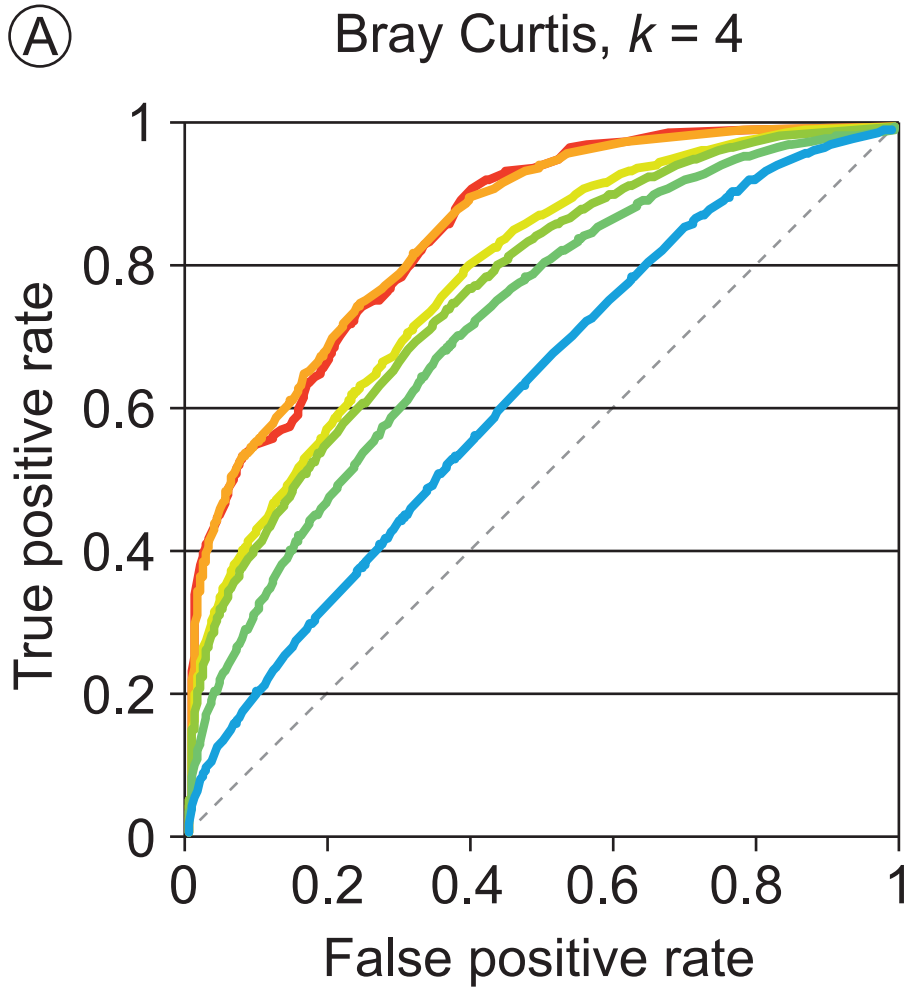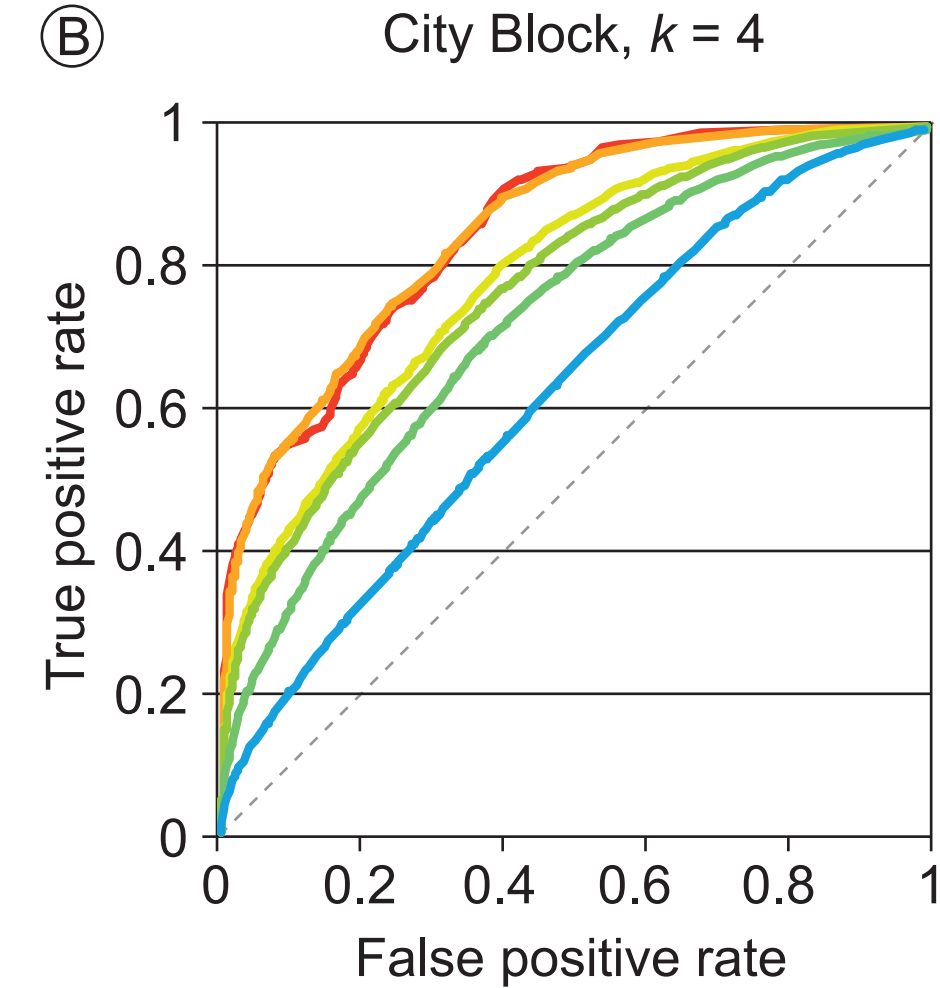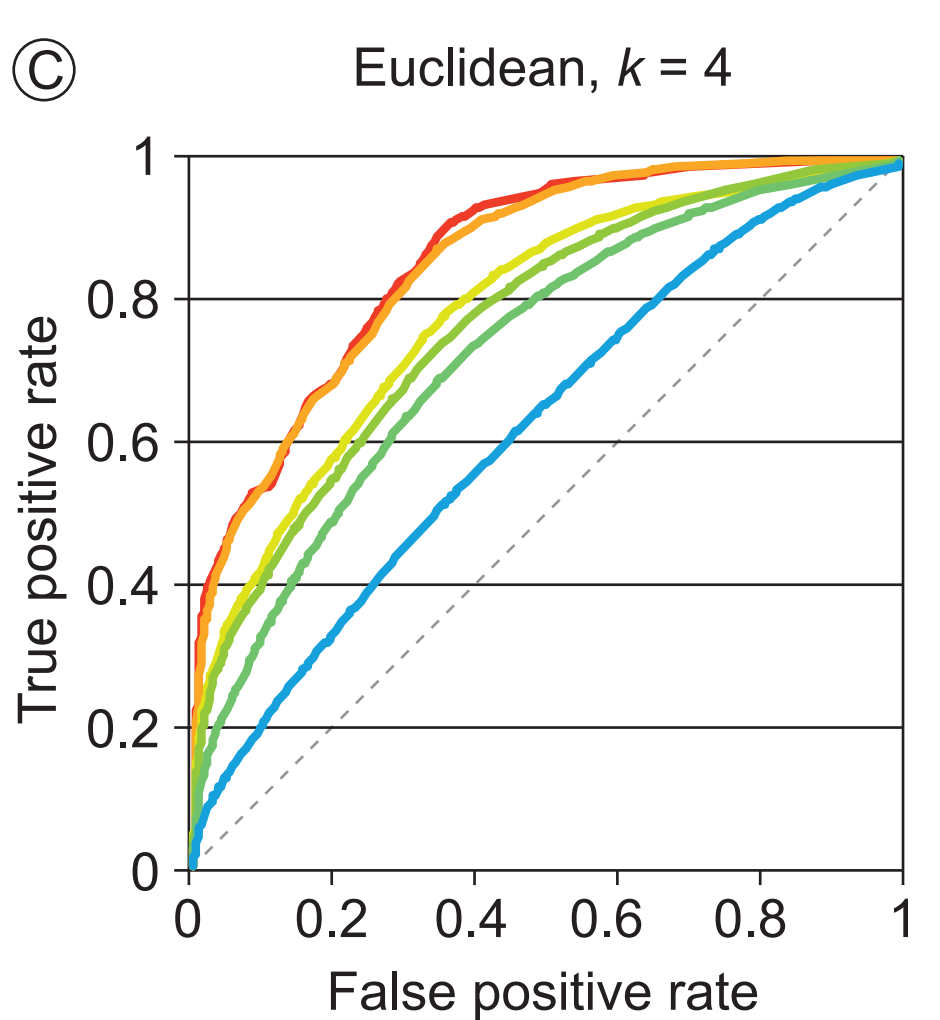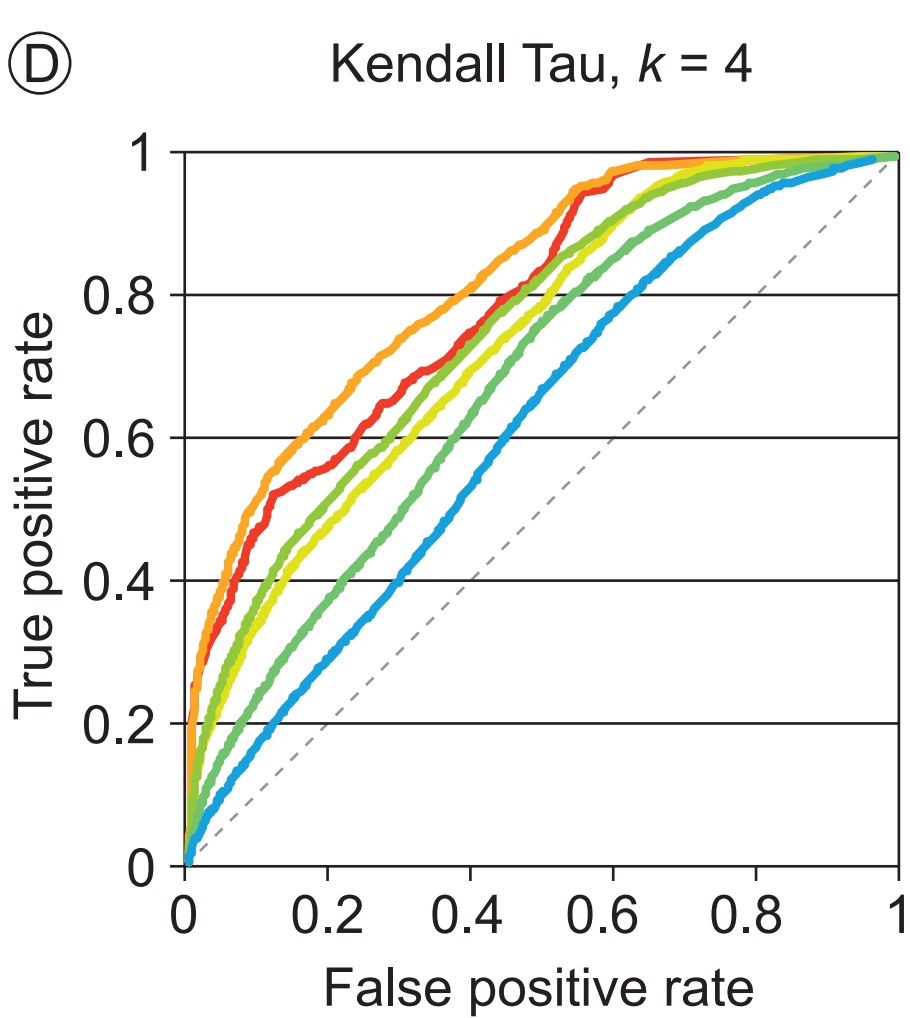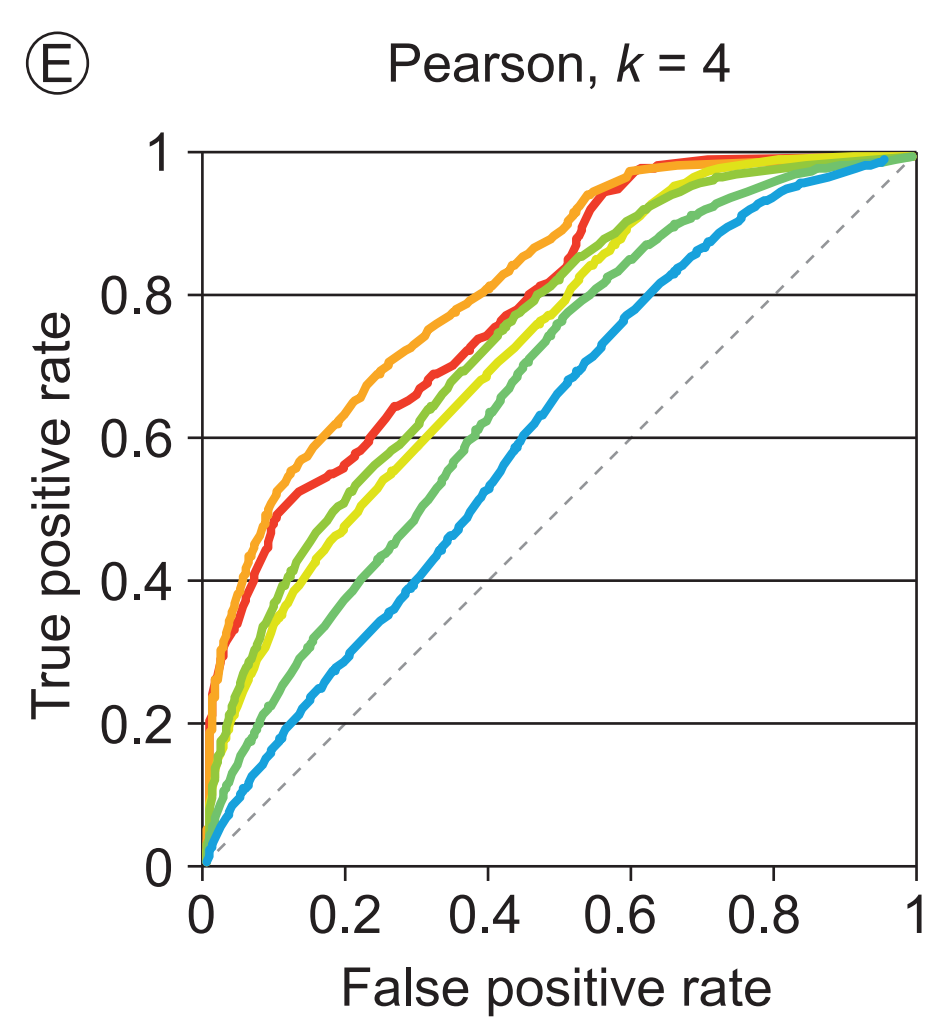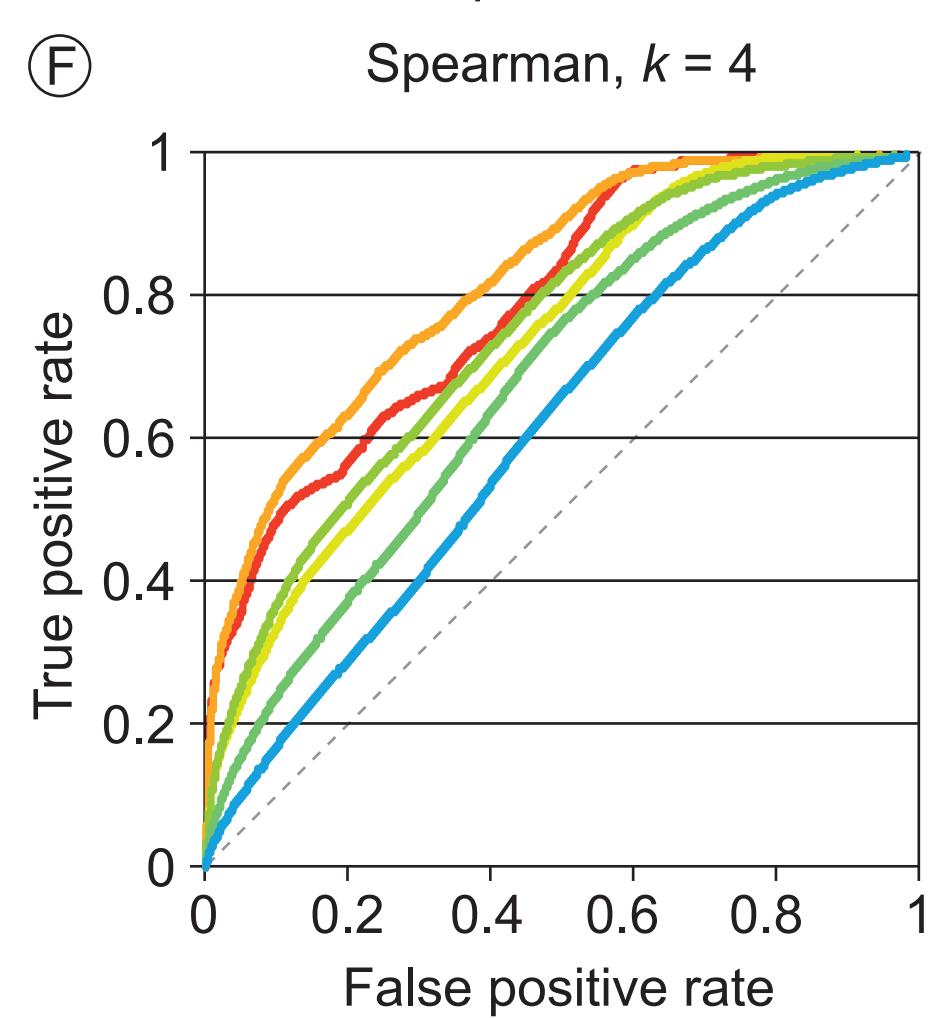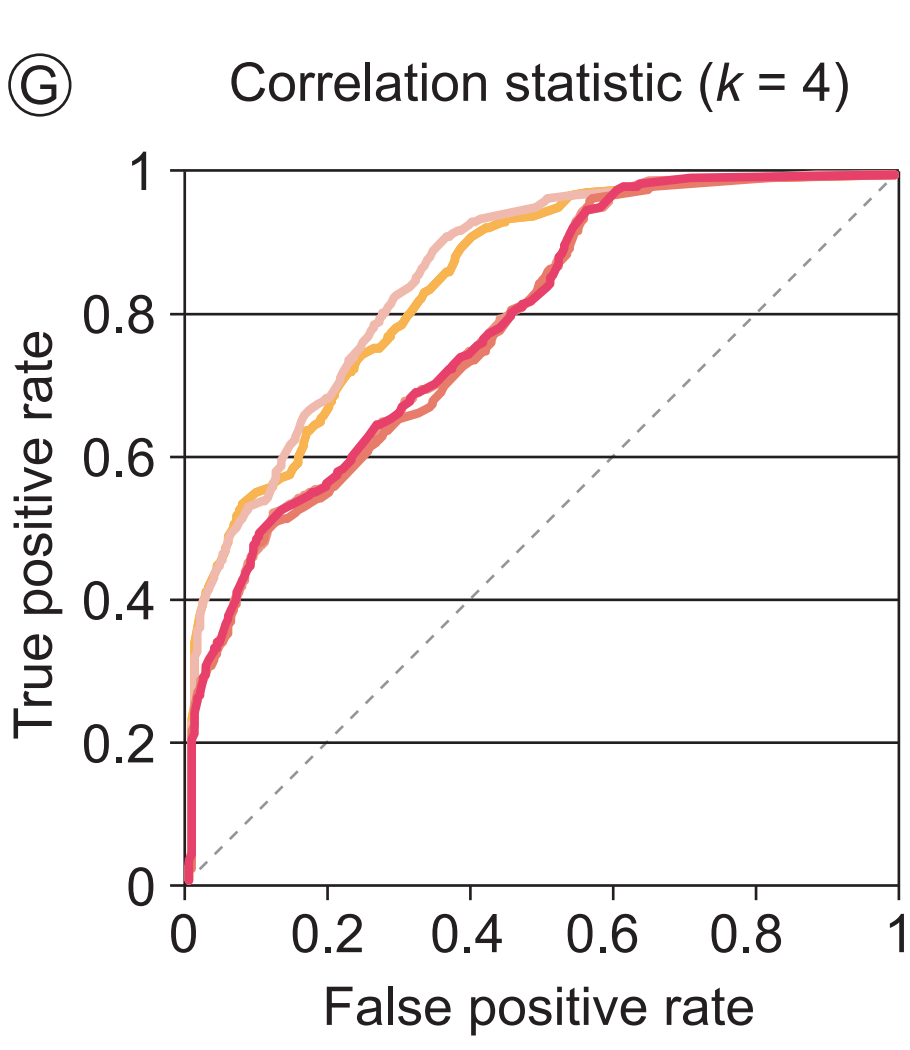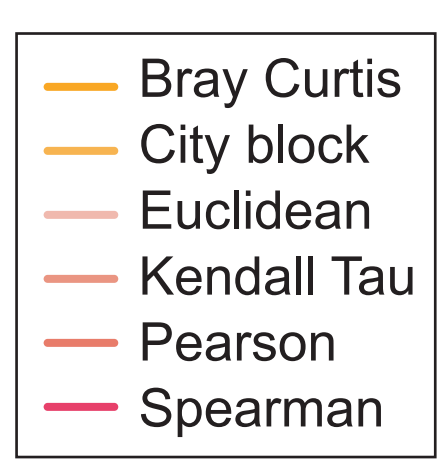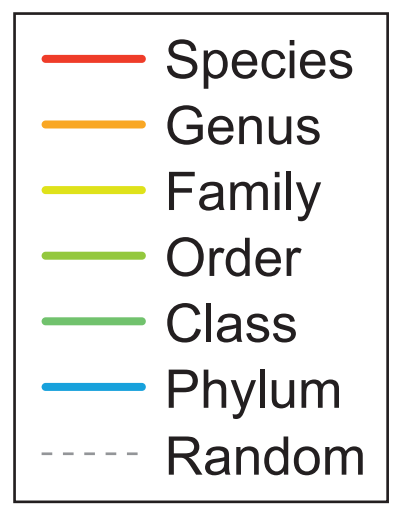

Supplement: Supplementary Data [file fuv048_supplementary_data.zip › SupplFigure_S2.pdf]

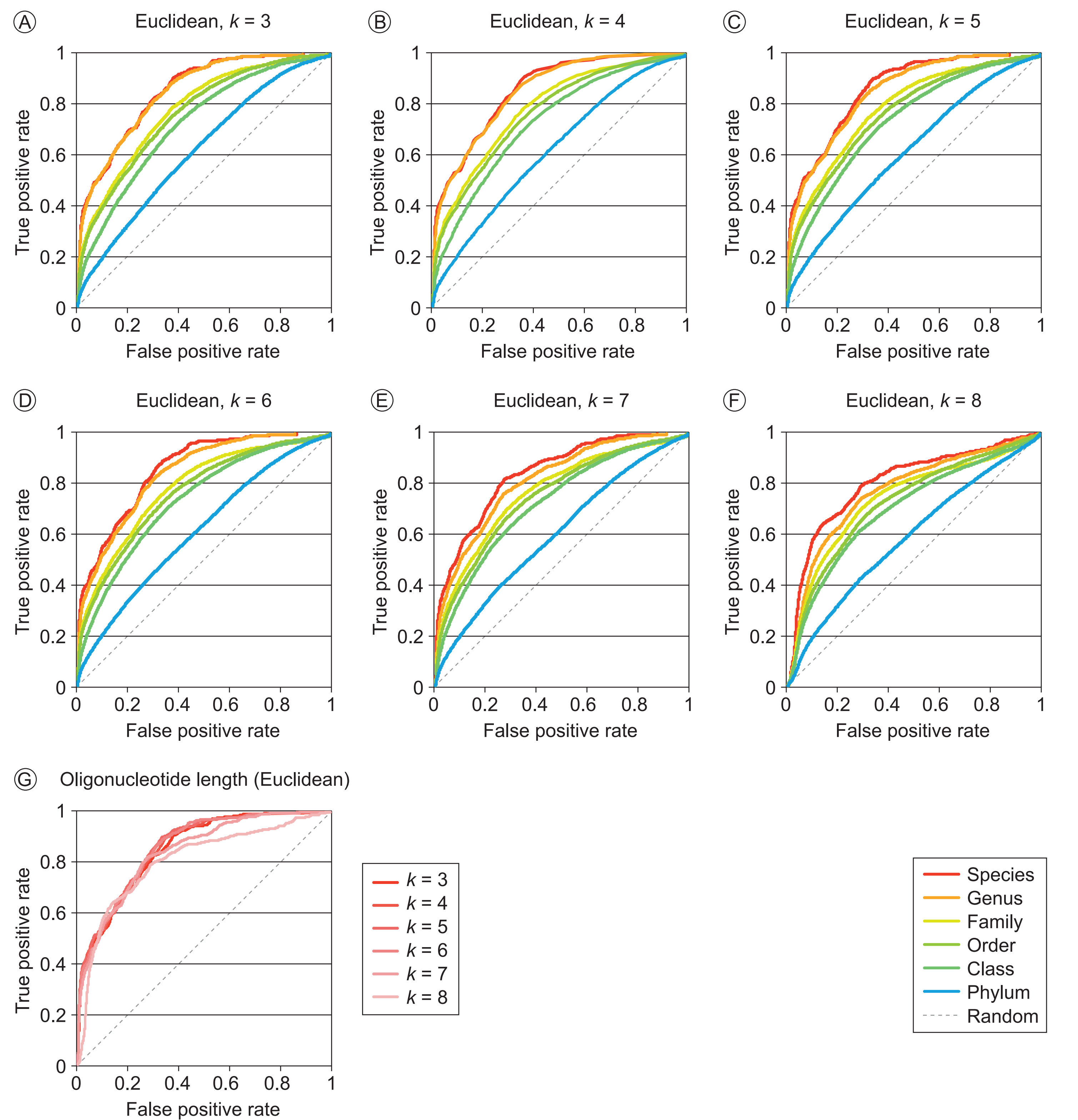

Supplement: Supplementary Data [file fuv048_supplementary_data.zip › SupplFigure_S3.pdf]

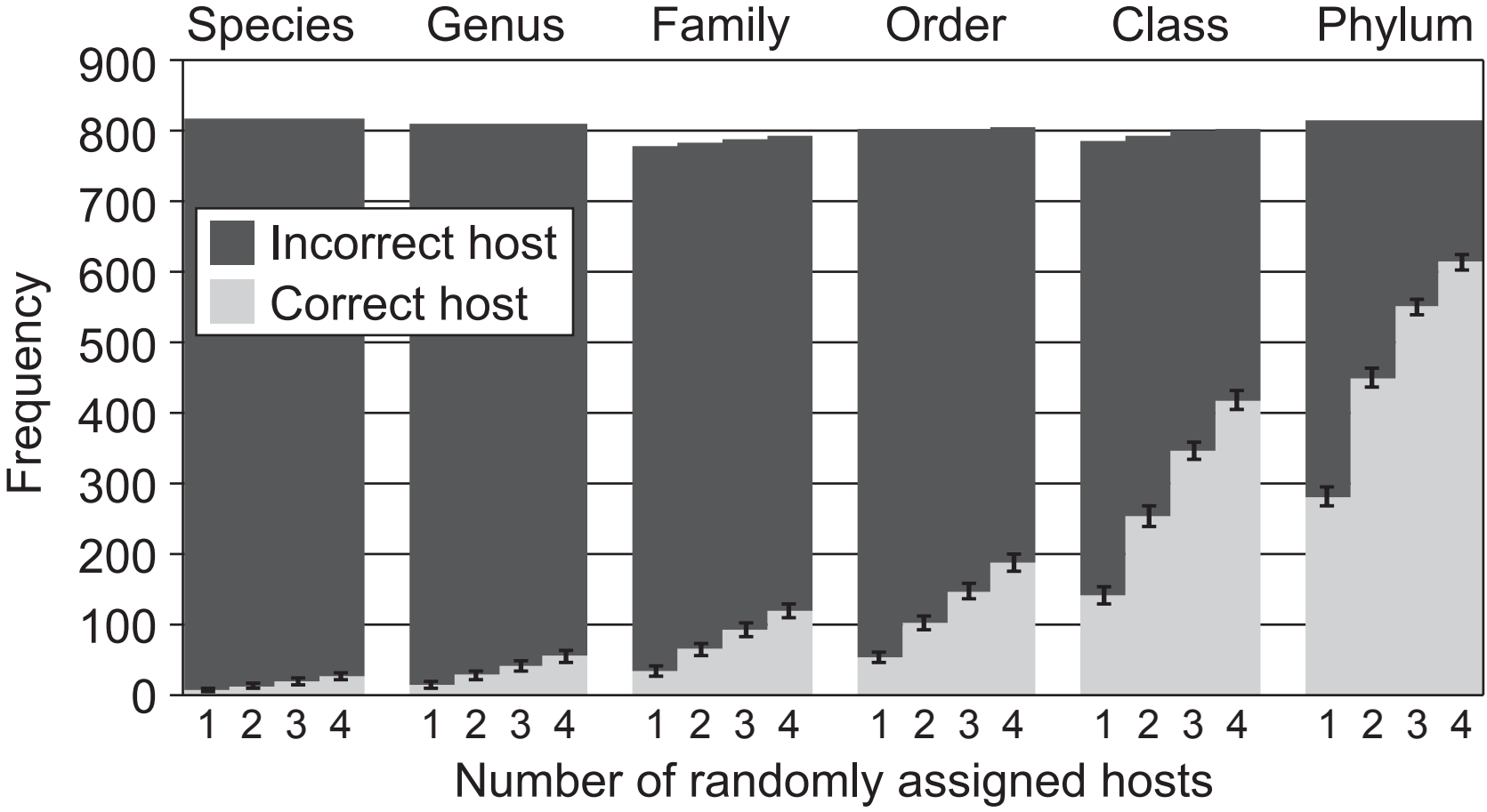

Supplement: Supplementary Data [file fuv048_supplementary_data.zip › SupplFigure_S4.pdf]
