# Supplementary material for: Computational approaches to predict bacteriophage–host relationships
Source: FEMS Microbiol Rev. 2015 Dec 9;40(2):258–72. doi: 10.1093/femsre/fuv048 (PMC5831537; doi:10.1093/femsre/fuv048)
Supplement: Supplementary Data [file fuv048_supplementary_data.zip › Supplement_figure _legends.doc]

*Supplemental Figure S1.* Histogram showing the environmental sources of 3,025 publicly available metagenomes included in the abundance profiling experiments.

*Supplemental Figure S2.* Receiver-operator characteristic (ROC) curves displaying the classification accuracy at taxomomic levels ranging from species to phyla of alternative correlation statistics of tetranucleotides for phage-host prediction: A) Bray Curtis; B) city block (Manhattan); C) Euclidean; D) Kendall; E) Pearson; F) Spearman; and G) all correlation statistics for *k*=4 at the species level.

*Supplemental Figure S3.* Receiver-operator characteristic (ROC) curves displaying the classification accuracy at taxomomic levels ranging from species to phyla of Euclidean distance in oligonucleotide usage profiles: A) trimers, *k* = 3; B) tetramers, *k* = 4 (see also Figure 1I); C) pentamers, *k* = 5; D) hexamers, *k* = 6; E) heptamers, *k* = 7; F) octamers, *k* = 8; and G) all lengths of *k* at the species level.

*Supplemental Figure S4.*  Number of phages with correct and incorrect hosts listed among 1-4 random hosts from the total dataset of 2,698 hosts (average ± standard deviation of 100 random samplings).
